# Nest-site competition and killing by invasive parakeets cause the decline of a threatened bat population

**DOI:** 10.1098/rsos.172477

**Published:** 2018-05-09

**Authors:** Dailos Hernández-Brito, Martina Carrete, Carlos Ibáñez, Javier Juste, José L. Tella

**Affiliations:** 1 Department of Conservation Biology, Estación Biológica de Doñana (CSIC), Avda. Américo Vespucio, 41092 Sevilla, Spain; 2 Department of Evolutionary Ecology, Estación Biológica de Doñana (CSIC), Avda. Américo Vespucio, 41092 Sevilla, Spain; 3 Department of Physical, Chemical and Natural Systems, University Pablo de Olavide, Ctra. de Utrera, km. 1, 41013 Sevilla, Spain; 4 CIBER of Epidemiología y Salud Pública (CIBERESP), Barcelona, Catalunya, Spain

**Keywords:** biological invasions, interspecific competition, impact, urban habitats

## Abstract

The identification of effects of invasive species is challenging owing to their multifaceted impacts on native biota. Negative impacts are most often reflected in individual fitness rather than in population dynamics of native species and are less expected in low-biodiversity habitats, such as urban environments. We report the long-term effects of invasive rose-ringed parakeets on the largest known population of a threatened bat species, the greater noctule, located in an urban park. Both species share preferences for the same tree cavities for breeding. While the number of parakeet nests increased by a factor of 20 in 14 years, the number of trees occupied by noctules declined by 81%. Parakeets occupied most cavities previously used by noctules, and spatial analyses showed that noctules tried to avoid cavities close to parakeets. Parakeets were highly aggressive towards noctules, trying to occupy their cavities, often resulting in noctule death. This led to a dramatic population decline, but also an unusual aggregation of the occupied trees, probably disrupting the complex social behaviour of this bat species. These results indicate a strong impact through site displacement and killing of competitors, and highlight the need for long-term research to identify unexpected impacts that would otherwise be overlooked.

## 1. Introduction

Biological invasions are considered as one of the most serious threats to biodiversity worldwide owing to their impacts on native biota and ecosystem functioning [1]. However, detecting and quantifying the effects of invasive species on native biodiversity can be challenging owing to the variety of potential, multifaceted impacts [1]. For example, invasive bird species may impact native species through different mechanisms such as predation, competition, hybridization or disease propagation [2]. Thus, significant research effort is required to assess each of these processes. Moreover, most research has focused on only a few invasive species. For example, the most recent review of studies on the impacts of invasive birds shows that published research focusing on potential impacts only exists for 18% of non-native bird species introduced worldwide [3].

The rose-ringed parakeet (*Psittacula krameri*) is one of the most studied invasive bird species, probably because it has established non-native populations worldwide [4–6] and is considered one of the 100 worst alien species in Europe (http://www.europe-aliens.org/speciesTheWorst.do). Thus, this species has been the subject of several reviews on ecological impacts [7,8] and of systematic risk assessments [9,10]. Some studies have assessed the negative impacts of rose-ringed parakeets on native birds through competition for food in bird feeders, where parakeets disrupt the foraging behaviour of native birds [11,12], and for nest sites of several cavity-nesting birds [13–19]. However, as in other invasive bird species for which information is available, these studies only indicate impacts on the individual fitness of native birds. More information is needed to properly assess impacts at a population level [3].

Some years ago, we reported an unexpected impact of rose-ringed parakeets (hereafter parakeets) on a threatened bat species, the greater noctule (*Nyctalus lasiopterus*, hereafter noctule) [16]. This is the largest European bat species and is classified as Vulnerable on the IUCN Red List of Threatened Species [20]. By 2003, the largest known European colony of this species was located in the María Luisa Park, an urban park located in Seville (southern Spain) where noctules used cavities of large trees as breeding sites and diurnal refuges. Ten years later (2013), we found several lines of evidence suggesting that the colonization of the park by invasive parakeets was having a negative impact on the noctule population: both species shared preferences for the same types of cavities, with noctules avoiding proximity to parakeets and parakeets occupying tree cavities previously used by noctules [16]. Here, we provide a longer-term monitoring study (until 2017), showing a dramatic reduction in the number of trees used by noctules linked not only to interspecific competition for tree cavities but also to aggressions by parakeets resulting in the death of noctules.

## 2. Material and methods

### 2.1. Study area and species

The María Luisa Park is situated in Seville, southern Spain (37°24′ N, 5°59′ W, altitude 10 m above sea level). With an area of approximately 40 ha, this park was established in 1850 and has dense vegetation, with large and old exotic trees (mainly *Platanus* sp., *Gleditsia triacanthos* and *Sophora japonica*) that offer numerous cavities for cavity-nesting species. There are no primary cavity-nesting species in this park (e.g. woodpeckers) that could excavate cavities, so all available cavities stem from tree decay. In addition, the land surrounding Seville is highly deforested and mostly devoted to agriculture, thus offering little habitat for forest-dwelling species.

The greater noctule has a very scattered distribution throughout central and southern Europe, and Spain constitutes the main core area for the species [20]. This species shows spatial sexual segregation [21] and, during the breeding season, females gather in small maternity colonies [20]. The María Luisa Park was thought to hold the largest local population of this species, as earlier studies conducted in just a sector of the park roughly estimated the presence of 500 adults (mostly females) [21]. Females form fission–fusion societies, resulting in several differentiated maternity colonies that occupy several cavities in different trees, with an average of 27 females simultaneously sharing a tree cavity [22,23]. Females may use different trees across the reproductive season and thus each maternity colony may occupy a minimum of 30 trees with adequate cavities, with the number of females using a particular cavity changing over time [22]. Nonetheless, females show long-term fidelity to the same groups of trees (some trees had been used for at least 14 years, [22]). Females usually arrive at the park in March, giving birth to one or two pups in May–June. Although most of them leave the park from August to November, others stay year round [21,22]. They have large foraging ranges, usually moving 15–40 km from the park in a single night to hunt insects and migrating songbirds [24]. Their annual survival rates were similar to those of other long-distance aerial-hawking bat species [25]. Genetically, this population is closely related to the two closest colonies of the species found in Jerez de la Frontera and Doñana National Park [26], located 60 km and 75 km from María Luisa Park, respectively.

The rose-ringed parakeet is a successful invader in Europe. Their success is a result of the large numbers of individuals imported as cage birds, frequent accidental escapes or deliberated releases and niche similarity between areas of introduction and their native Asian habitats [27,28]. The first introduction in Seville most probably occurred in 1992 with a very small group of individuals deliberately released in María Luisa Park. This is a highly sociable species that nests in cavities (mostly in trees but also in buildings, [16]) and produces one to four offspring per year. In María Luisa Park, most parakeets select tree cavities for nesting between late December and February and lay eggs mostly in March. There is, however, large variation in breeding phenology among pairs, with fledglings leaving the nests between May and early July. Moreover, newly formed immature pairs and groups of non-breeding individuals inspect and use tree cavities throughout the breeding season.

Another eight cavity-nesting bird species are breeding in María Luisa Park [16]. However, we have previously shown that the only species competing with noctules for tree cavities was the rose-ringed parakeet [16]. Thus, the rest of the species were not considered in this study.

### 2.2. Population monitoring

Although our first studies on noctules in María Luisa Park date back to 1992 [22], it was not until 2003 that we geolocated all trees used by the species in a single year. Unfortunately, we were not able to obtain a census of the whole adult population of noctules. Obtaining accurate population sizes of forest bats forming fission–fusion societies, such as the greater noctule, is extremely difficult [29,30]. In fact, there is no published information on population sizes for this or similar bat species. Therefore, we relied on temporal changes in the number of trees used by the species as a surrogate of changes in population size. We did not know the breeding population size of parakeets in 2003 as the species was still very scarce and little attention was paid to it because no impacts were expected at that time. We can, however, obtain a reliable estimate of the number of parakeet nests in 2003 from a demographic model built with the year of the first introduction, detailed population counts conducted since 2011 and breeding parameters obtained from the same population [31].

In 2013 and 2016–2017, we simultaneously took a census of the number of nests of parakeets and the number of trees used by noctules in the park. Our monitoring programme extended from January to early August (covering the breeding season of both species), and observations were conducted in the morning (08.00–12.00 h) and afternoon (17.00–21.00 h). We GPS-located (±3 m) each year all tree cavities that we were able to visually inspect using 10 × 50 binoculars. We visually estimated the height of the cavity above ground (in m) and the width of its entrance (in cm), which was scored as small, medium or large (less than 4 cm, 4–8 cm and greater than 8 cm, respectively) [16]. We did not consider the orientation of the cavities [16], as in other studies on nest-site competition between parakeets and native species [14,15,17–19], because orientation did not affect the selection of cavities in a previous study [13]. Our previous analyses showed a preference of both parakeets and noctules for cavities well above ground (approx. 15 m) and with medium-sized entrances [16]. Thereafter, we repeatedly visited and observed at a distance (for a minimum of 10 min) each cavity on at least 10 different days evenly spaced throughout the breeding season of the two species. Given that parakeets can enter cavities that are not used as nests (see above), we conservatively considered as active parakeet nests those cavities where we observed adults entering a minimum of 10 times on different days, heard chicks inside or observed juveniles at the entrance. Regarding the identification of trees used by noctules, we complemented the previous methodology for monitoring tree cavities with the use of an ultrasound bat detector (Pettersson D230) and systematic observations at sunset to observe noctules leaving tree cavities [16].

### 2.3. Aggressive interactions

Interactions between parakeets and noctules were observed during the monitoring activities described above. When we observed harassment and attacks of parakeets towards noctules present inside or in the entrance of their tree cavities, we recorded the duration of the aggression (in minutes), whether the noctule was expulsed and whether the parakeet later entered the cavity. Dead and injured noctules were also found, always under trees occupied by noctules. After unexpectedly encountering the first case of a dead noctule, we proceeded to record these events more systematically. We are convinced that many cases were overlooked because dead and injured noctules could be hidden by ground vegetation, scavenged by cats and rats or, as we later learned, recovered by people working in the park or visitors. The corpses we found were transported to the laboratory for a detailed visual examination of damage to the skin and bones.

### 2.4. Spatial arrangement of noctules

Each year (2013, 2016 and 2017) we geolocated the cavities used by parakeets and noctules as well as those that remained unoccupied. The occupancy of a particular tree cavity by noctules could be influenced by the availability of unoccupied cavities as well as by the spatial distribution of cavities occupied by the same and/or other species, driven not only by competition but also by conspecific attraction processes [16]. We thus measured the Euclidean distance from each cavity to the nearest cavity occupied by noctules and parakeets (nearest-neighbour distance) as well as the corresponding aggregation indexes. Aggregation indexes were obtained as the relative position of each cavity within the whole distribution of all cavities occupied by conspecifics or heterospecifics using Σexp(−*d_ij_*), with (*i* ≠ *j*), where *d_ij_* is the linear distance between cavities *i* and *j*, and *j* represents all occupied cavities [32]. Nearest-neighbour distances and aggregation indexes are complementary and depict the social environment around each cavity at a landscape scale as well as the existence of close conspecifics and competitors in its proximity [16]. The two main sources of habitat heterogeneity in our study area, i.e. the proximity to surrounding streets and forest cover, were not considered because our previous work showed no effects on the spatial arrangement of noctules and parakeets [16].

We employed generalized linear models (GLMs) with a binomial error distribution and logistic-link function to ascertain factors explaining the probability of occupation of a cavity by noctules, fitting as explanatory variables its traits (entrance size and height above ground) and its nearest distance and aggregation to both noctules and parakeets. As values of nearest distance and aggregation for the same species were highly correlated (all *p* < 0.001), we alternatively included in models only one of these spatial descriptors. Continuous variables were standardized for modelling. Models were separately built for the most distant years (2013 and 2017) from which we gathered spatial information from both species, using the Akaike information criterion corrected for small sample sizes (AICc) for model selection [33]. Within each set of candidate models, we calculated ΔAICc*i* as the difference between the AICc of model *i* and that of the best-supported model (i.e. the model with the lowest AICc). Models within 2 AICc units of the best supporting model were considered as alternatives. We also quantified the plausibility of each model as being the best approximation using Akaike weights, *w* [33], and performed model averaging (MuMIn package) to estimate the relative importance of all variables through the calculation of model-averaged estimates and confidence intervals (CIs) using the set of alternative models. A given effect received no, weak or strong support when the 95% CI for the coefficient estimate strongly overlapped zero, barely overlapped zero or did not overlap zero, respectively. We calculated the percentage of deviance explained by the best-supported models, obtained as 100 − (deviance of model *i*/deviance null model) * 100), for assessing their goodness of fit. All statistical analyses were conducted in R v. 3.1.2 [34], and raw data used for analyses are provided in the electronic supplementary material.

## 3. Results

### 3.1. Interspecific aggressions

**Figure 1.**
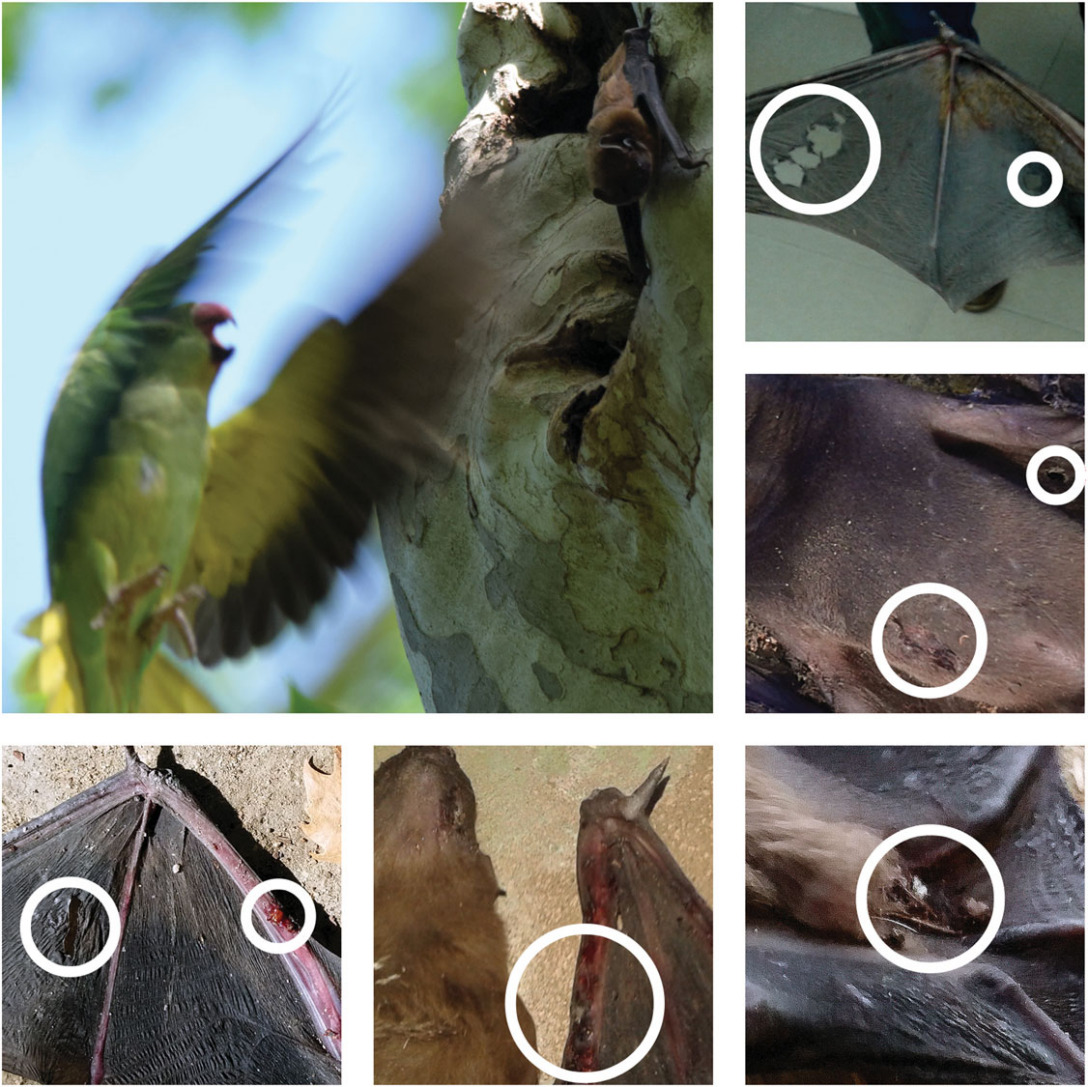
Main picture: an adult female rose-ringed parakeet attacks a greater noctule at the entrance of a tree cavity used as a refuge by noctules in María Luisa Park, Seville, on 17 May 2016. One noctule was found dead under the same tree the next day, with wounds caused by parakeets. Accompanying pictures: details of different types of wounds caused by parakeets on noctules found dead under their tree refuges (photographs: Dailos Hernández-Brito).

During the breeding seasons of 2016 and 2017, we recorded 36 aggressions of parakeets towards noctules in trees occupied by both species. Most aggressions (55.6%) were recorded in May and during the 3 h before sunset (83.3% of aggressions). All of these aggressions happened at the entrance of tree cavities, and in 16 cases, we observed parakeets attacking noctules with their beaks while emitting loud sounds (figure 1). These aggressions usually lasted from 1 to 25 min, reaching in one case up to 145 min (median: 13.13 min), and ended with the parakeets entering the tree cavity after the attacked noctule was expulsed and obliged to flee during the daytime. Once parakeets entered the tree cavity, we were not able to observe whether or not they attacked other noctules that may have been inside the cavity. In 20 other instances, parakeets ceased their aggression and left the tree at sunset without successfully expelling the noctules from their refuge cavities.

During the same period, we found 20 dead and two injured noctules under 18 different trees, all of them with active nests of parakeets. Seven of these noctules were found dead under the same tree where we recorded aggressions by parakeets the previous day. Dead noctules included three few-day-old pups, 10 lactating young and seven adults, as well as one pregnant female. Eight of these corpses were too putrefied to allow us to observe wounds or other indications of attack (temperatures reached 33–40°C during the study period, so corpses decomposed rapidly). The other 12 corpses were fresh enough to show clear wounds caused by the beaks of parakeets (figure 1). These wounds were present mainly on the wings, consisting of holes in the membranes (dactylopatagium and plagiopatagium areas), chafing on the skin covering the phalanges and forearm, breakages of phalanges, and wounds both on the upper side of the body and the abdomen. The two injured, but still living adult noctules showed similar wounds and were not able to fly or to climb the trunks of trees to return to their cavity refuges. We suspect that noctules were injured and killed when parakeets entered their cavities after expelling other noctules (see above), because pups and lactating young do not leave the cavities. We did not observe aggressions addressed to noctules by other species, neither did we find evidence of other species preying on or killing noctules in the park.

### 3.2. Temporal trends in the occupation of trees

**Figure 2.**
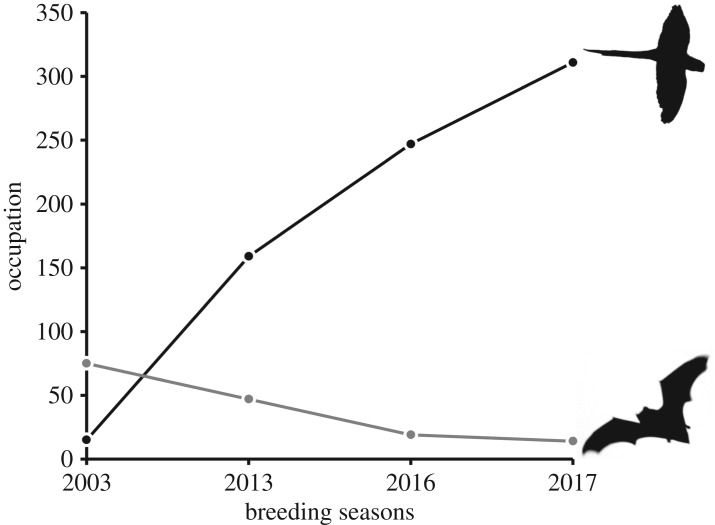
Changes in the number of nests of rose-ringed parakeets and trees occupied by greater noctules across years in María Luisa Park.

Figure 2 shows the numbers of nests of parakeets and trees occupied by noctules in María Luisa Park from 2003 to 2017. Parakeets were scarce in this area in 2003 and, although we did not conduct a detailed census, our demographic-based estimation suggests that the local breeding population would have been as low as 13 nests at that time. Three annual detailed censuses conducted since 2013 showed an increase to 311 active nests in 2017 (figure 2). This means a 96% population increase from 2013 to 2017, and an estimated 2192% population increases from 2003 to 2017. Noctules showed an opposite trend: cavities used as refuges were found in 75 trees in 2003, the number decreasing to only 14 trees in 2017 (figure 2). This resulted in a 70% decrease between 2013 and 2017, and an 81% decrease between 2003 and 2017.

### 3.3. Spatial segregation between noctules and parakeets

**Figure 3.**
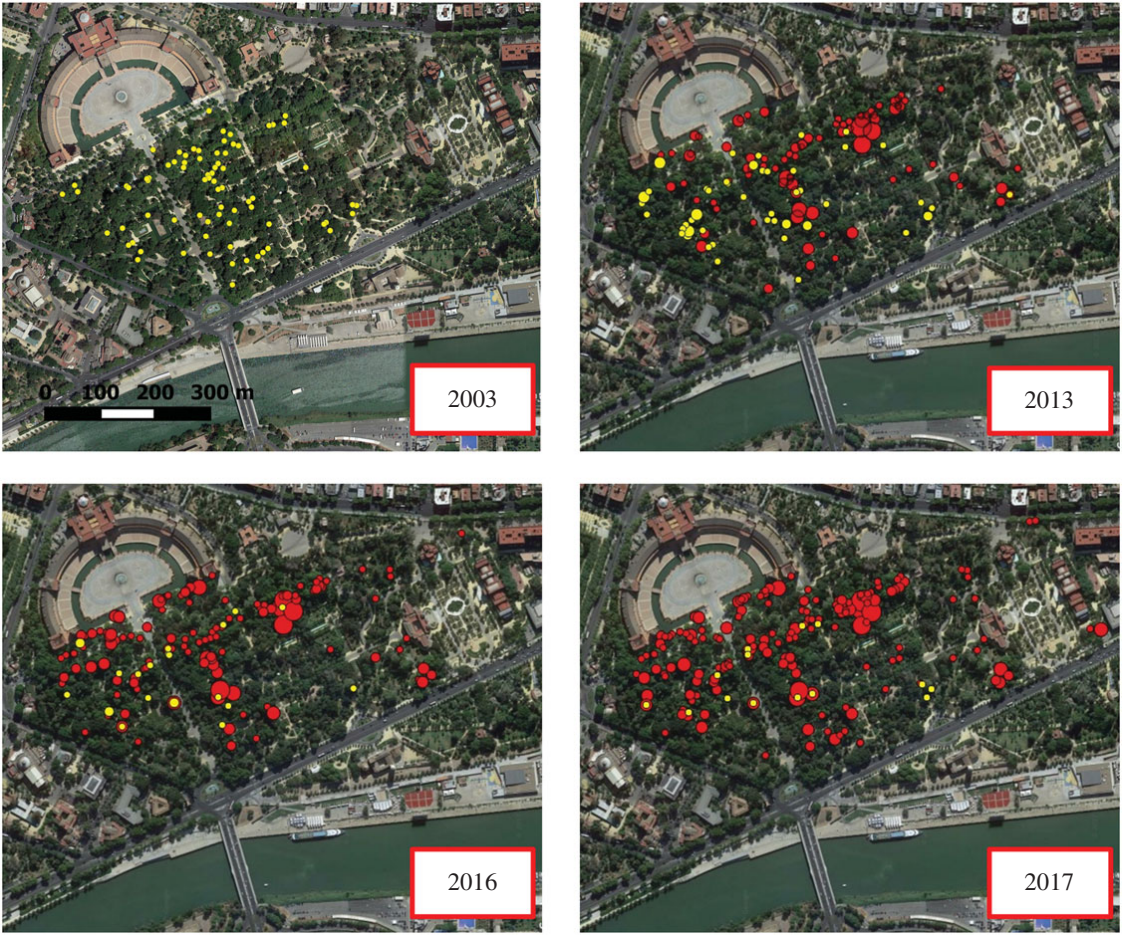
Distribution of trees with refuges of greater noctules (yellow dots) and with nests of rose-ringed parakeets (red dots) in María Luisa Park across years. Larger dots indicate overlapping points.

Figure 3 shows the spatial distribution of parakeet nests and noctule refuges recorded across years in the park, indicating not only a reduction in the number but also a spatial contraction of noctule refuges along with an increase and spatial expansion in parakeet nests. In 2013, the first year we recorded both noctule refuges (*n* = 47 trees) and parakeet nests (*n* = 159 nests) (figure 3), the best-supported model indicates that the probability a tree cavity was occupied by noctules was related to its entrance size and height above ground and to the nearest distances to other noctule refuges and parakeet nests (table 1). In this year, noctules used cavities well above ground level and with medium- to large-size entrances, close to other noctule refuges and far from the nearest parakeet nest (table 2). In 2017, when the number of trees occupied by noctules had declined to 14 while the number of parakeet nests had increased to 311 (figure 3), the four alternative models obtained for the probability of occupation of cavities by noctules also included variables describing the characteristics of the cavity as well as the descriptors of the presence of parakeet and noctules in the surrounding area (table 1). However, after model averaging, the only variable receiving strong support was the distance to the nearest parakeet nest (table 2). Thus, the probability of occupancy of a cavity by noctules was higher than expected at short distances to parakeet nests. These results suggest that in 2017, noctules were forced to use cavities at any height above ground or size, being unable to avoid close proximity to some parakeet nests (most of the trees used also held a parakeet nest; see below).

**Table 1.** GLMs obtained to explain the probability of occupancy of tree cavities by greater noctules in María Luisa Park, Seville, in 2013 and 2017. (Explanatory variables reflect the size of the cavity entrance (size; small size taken as reference), the height above ground of the cavity entrance (height), the distance to the nearest noctule refuge (nndn) or parakeet nest (nndk) and the aggregation of noctule refuges (agregn) and parakeet nests (agregk) around each cavity. Only the 10 first models, after ranking using AICc, are shown (null models were ranked 34th and 21st for 2013 and 2017, respectively). Models with ΔAICc less than or equal to 2 were considered as alternative (in italics). *K*: number of parameters; AICc: Akaike information criterion corrected for small sample sizes; ΔAICc: difference between the AICc of model *i* and that of the best-supported model (i.e. the model with the lowest AICc); weight: Akaike weights; %dev: deviance explained by alternative models.)

models 2013	*k*	AIC	ΔAICc	weight	%dev
*size, height, nndk, nndn*	*6*	*302*.*7*	*0*	*0*.*79*	*22*.*61*
size, height, agregk, nndn	6	306.1	3.38	0.15	
size, height, nndn	5	307.9	5.16	0.06	
height, nndk, nndn	4	314.9	12.13	0.00	
height, agregk, nndn	4	317.2	14.48	0.00	
size, nndk, nndn	5	318.2	15.5	0.00	
size, agregk, nndn	5	319.5	16.7	0.00	
height, nndn	3	319.5	16.8	0.00	
size, nndn	4	320.5	17.74	0.00	
agregk, nndn	3	332	29.2	0.00	

models 2017	*k*	AIC	ΔAICc	weight	%dev
*size, nndk*	*4*	*124*.*9*	*0*	*0*.*33*	*17*.*76*
*size, nndk, agregn*	*5*	*125*.*5*	*0*.*58*	*0*.*24*	*18*.*79*
*size, height, nndk*	*5*	*125*.*9*	*0*.*96*	*0*.*20*	*18*.*52*
*size, height, nndk, agregn*	*6*	*126*.*6*	*1*.*66*	*0*.*14*	*19*.*47*
nndk	2	129.3	4.42	0.04	
nndk, agregn	3	130.4	5.46	0.02	
height, nndk	3	130.6	5.72	0.03	
height, nndk, agregn	4	131.8	6.87	0.01	
size, height, agregn	5	137.7	12.8	0.00	
size, height	4	138.1	13.16	0.00

**Table 2.** Variables explaining the probability of occupancy of tree cavities by greater noctules in María Luisa Park, Seville, in 2013 and 2017. (Averaged estimates and 95% CIs (2.5% and 97.5%) were obtained from the best-supported models (ΔAICc less than or equal to 2, table 1). The effect of a given variable has no, weak or strong support when the 95% CI strongly overlapped zero, barely overlapped zero or did not overlap zero, respectively. Table 1 gives abbreviations of variables.)

variables	estimate	2.50%	97.50%
2013			
intercept	−5.90	−7.58	−4.58
medium size	1.89	0.8	3.35
large size	2.06	0.89	3.56
height	0.62	0.33	0.92
nndk	0.62	0.17	1.06
nndn	−3.11	−4.51	−1.91
2017			
intercept	−1.71	−23.85	20.42
medium size	1.37	−0.82	3.56
large size	2.26	−0.18	4.70
nndk	−4.74	−8.82	−0.66
agregn	5.65	−14.67	25.97
height	0.09	−0.28	0.46
agregk	0.00	−0.10	0.10

The occupancy of trees by noctules may also be related to temporal changes in the availability of trees with adequate cavities because of tree decay and management of the park by public garden authorities. However, our previous results showed that 49 of the trees occupied by noctules in 2003 were unoccupied in 2013 by this species, despite the fact that they still had cavities (which were occupied by parakeets in almost half of the cases), and that the probability of tree abandonment was positively related to the presence of parakeet nests in the same tree and to the aggregation of parakeet nests around the tree (see [16] for statistical analyses). Only two of the 47 trees occupied by noctules (4.25%) in 2013 were also occupied by parakeets. In 2017, 38 trees previously occupied by noctules were unoccupied by this species but occupied by parakeets. In contrast to 2013, 11 of the 14 trees occupied by noctules (78.6%) were also occupied by parakeet nests.

## 4. Discussion

Detecting the negative effects of invasive species on native biodiversity is challenging. While threats to endangered, endemic or charismatic species generally attract attention, gradual changes in abundance and distribution of common species tend to pass unnoted [1]. Moreover, little attention has been paid to the potential impacts of non-native species invading urban environments because it is often assumed that these habitats hold low biodiversity, which is mainly represented by common, widespread human commensal species, leaving vacant niches for invaders [35]. Here, we demonstrate a process by which an invasive bird species, mostly occupying urban environments [28], impact a threatened bat species. These impacts would have passed unnoted if the bat population had not been monitored 14 years ago, previous to the spread of the invader.

Our results, together with previous knowledge on the behavioural ecology of this population of noctules, allow us to hypothesize that a temporal process exists in their interactions with parakeets. The long-term monitoring (1992–2006) of transponder-equipped and radio-tagged noctules showed that they returned year after year to the park, forming a complex fission–fusion society. Females often switched roosting trees but differentiated maternity colonies remained constant both spatially and across years [22,23]. On the other hand, parakeets are highly aggressive against other cavity-nesting species and even against predators of much larger size, winning most of the aggressive encounters that we have recorded [16]. Thus, during the first 10 years of this study (2003–2013), the slow but progressive increase in parakeet numbers may have caused an increase in the number of noctule cavities occupied by parakeets when noctules arrived at the park in March. In addition, some noctules were actively expulsed from their cavities by parakeets. This surely forced noctules to look for alternative cavities far from the trees occupied by parakeets. By 2013, the number of trees occupied by noctules was reduced by 30%, and these trees were spatially aggregated and segregated from the proximity of parakeet nests. There is the possibility that some noctules dispersed to other colonies owing to the progressive scarcity of tree cavities and harassment by parakeets. The two closest colonies are located in Doñana National Park and Jerez de la Frontera, 60 and 77 km from María Luisa Park, holding a maximum of 40 and 150–200 individuals across years, respectively. Although genetic studies showed no differentiation between these colonies [26], our long-term monitoring of the three colonies does not support this idea. While many of the more than 300 females marked in María Luisa Park were recaptured within the same park across years [22], only one of these females visited Doñana (in 2001, 2003 and 2004), while two females marked in Doñana and Jerez visited María Luisa Park (2007 and 2017) and three females marked in Jerez visited Doñana (2004–2016), all for short periods of time. These data indicate that contacts between colonies seem to be occasional and performed by very few individuals. In addition, the decrease of the María Luisa Park population was not paralleled by any drastic increase in the other two colonies. A further population increase in parakeets would have led to a greater reduction and spatial contraction of noctule tree refuges in María Luisa Park. By 2017, the number of cavities not used by parakeets could have been so low that noctules were forced to use any available cavity in trees that hold parakeet nests, in almost 80% of cases. It is worth noting that the deviance explained by occupancy models was relatively low (table 2), probably because many of the unoccupied cavities included in the statistical analyses could be, in fact, unsuitable for the species. (We could not inspect the interior of unoccupied cavities and thus we did not know whether they were large enough to hold parakeet nests and noctule refuges.) This extreme competition for tree cavities and the close proximity of parakeet nests could explain the aggressive attacks and killing of noctules by parakeets recorded in 2016–2017. However, many dead noctules could have passed unnoted (see Material and methods), and these aggressions may have been overlooked in the past. In fact, an aggression had been reported in 2005 (cited in [16]), and several people working in María Luisa Park spontaneously told us in 2016 that they frequently observed aggressions by parakeets and recovered dead and injured noctules in past years. Therefore, it seems that aggressions and fatal attacks do not constitute a recent phenomenon but perhaps increased in frequency and intensity as a result of the increased competition for cavities, thus making observation more likely by researchers.

In contrast with a recent review of impacts by invasive bird species [3], our results indicate an impact not only on individual fitness of native fauna (i.e. through nest-site usurpation and displacement to presumably poorer sites) but also at the population level, which has been thus far poorly studied. Greater noctules, weighing approximately 50 g, are unique because they are the only bat species in Europe that are able to hunt small passerine birds (usually less than 25 g) while they are migrating at night at long distances from their maternity colonies [36,37]. However, noctules do not seem to be able to survive the attacks of the much larger and more powerful parakeets (weighing approx. 120 g, figure 1). In fact, parakeets have been observed killing other bird [38] and bat species [39], and even rats [40]. Therefore, the 81% reduction in the number of trees occupied by noctules in 14 years seems to be the result of both site displacement and direct mortality caused by parakeets. Bats are long-lived species with slow reproduction rates, and thus, their ability to compensate for high predation rates is very limited [41]. Moreover, the annual survival rates of noctules living in María Luisa Park before the population growth of parakeets was low (0.74) compared to other forest bat species that forage at shorter distances [25]. Therefore, an increment in mortality rates caused by parakeets both on lactating and adult noctules would severely affect the population dynamics of this population and contribute to its dramatic decline. Unfortunately, we were unable to measure temporal changes in population size owing to the methodological difficulties of accurately estimating population sizes of forest bat species that form fission–fusion societies [29,30]. However, it is reasonably expected that the population size would have declined in parallel with the 81% reduction in the number of tree cavities used. Population size, as well as social structure, is expected to be affected: females were originally distributed across the park forming differentiated, stable maternity colonies ([22,23]; figure 3), something certainly disrupted given the scarcity and aggregation of the trees currently used (figure 3). This social disruption may have unexpected effects on the breeding biology of the species.

Actions to reduce the population of parakeets and to provide artificial refuges for noctules are urgently needed. Without that, this population, which was once the largest known for this threatened bat species in its range [23], could be completely extinct within a few years. The provision of artificial refuges alone would be insufficient, given that noctules learn to use them slowly (C. Ibáñez 2003, unpublished data) and that the population size and direct impacts of parakeets are increasing at a much faster rate. An eradication plan of parakeets was planned for early 2017 by the city government of Seville, but this was cancelled owing to pressures by animal welfare associations, even though it was supported by a Spanish law which specifically deals with the management of invasive species (Real Decreto 630/2013). Programmes for the control or eradication of invasive species often face public opposition [42], especially when dealing with charismatic species such as parakeets [43].

Our work exemplifies the challenges in understanding the true ecological impacts of invasive birds. While the number of introduced non-native bird species increases worldwide [44] and invasion risks increase in new regions [5], the scientific community is only able to study a small fraction of these populations and their multifaceted potential impacts [3]. Moreover, some impacts, such as those reported here, are unexpected and can be easily overlooked in the absence of long-term research. In this sense, we speculate whether other impacts on bats remain hidden. Many European bat species, some of them threatened, are closely linked to buildings and urban habitats [45–47]; meanwhile, parakeets are spreading across European cities [27,28]. There is concern that parakeets could reduce the availability of suitable tree cavities for the noctule bat (*Nyctalus noctula*) in the Netherlands [48]. One Leisler's bat (*Nyctalus leisleri*) was found killed by parakeets in Italy [39], and parakeets seem to be competing for greater noctule refuges in Jerez de la Frontera (I. Sánchez and D. Hernández-Brito 2017, personal observation). Parakeets can also compete for cavities in buildings that they use for nesting, which can be usurped from other bird [16] and bat species. One of the authors (D. Hernández-Brito) observed in Seville, on 7 July 2017, three parakeets attempting to force isabelline serotine bats (*Eptesicus isabellinus*) out from a wall cavity in a tall building during the daytime. Seventy-eight bats later flew out from that cavity at sunset. All of these observations can be considered as anecdotal and do not necessarily imply an impact on bat populations. Similarly, the first observation of parakeets harassing a noctule in María Luisa Park in 2005 [16] would not have indicated a population impact until the long-term research presented here. As a matter of concern, a recent meta-analysis shows that large impacts caused by invasive species can often be missed owing to small sample sizes, resulting in high Type II error rates and false certainty of no impact [49]. Therefore, much more research is needed to properly assess the impact of parakeets and other invasive species on a variety of bat species [50].

## Supplementary Material

Explanatory variables for occupation of cavities by noctules

## Data accessibility

The dataset used for analysing the occupation of cavities by noctules has been uploaded as the electronic supplementary material. The rest of the raw data are provided in the body of the article

## References

[1] Simberloff D et al. 2013 Impacts of biological invasions: what's what and the way forward. Trends Ecol. Evol. 28, 58–66. (doi:10.1016/j.tree.2012.07.013) 22889499 10.1016/j.tree.2012.07.013

[2] Blackburn TM, Lockwood JL, Cassey P 2009 Avian invasions: the ecology and evolution of exotic birds. Oxford, UK: Oxford University Press.

[3] Martin-Albarracin VL, Amico GC, Simberloff D, Nuñez MA 2015 Impact of non-native birds on native ecosystems: a global analysis. PLoS ONE 10, e0143070 (doi:10.1371/journal.pone.0143070) 26576053 10.1371/journal.pone.0143070 PMC4648570

[4] Butler CJ 2003 Population biology of the introduced rose-ringed parakeet *Psittacula krameri* in the UK. PhD thesis, University of Oxford, Oxford, UK.

[5] Cardador L, Lattuada M, Strubbe D, Tella JL, Reino L, Figueira R, Carrete M 2017 Regional bans on wild-bird trade modify invasion risks at a global scale. Conserv. Lett. 10, 717–725. (doi:10.1111/conl.12361)

[6] Pârâu LG et al. 2016 Rose-ringed parakeet *Psittacula krameri* populations and numbers in Europe: a complete overview. Open Ornithol. J. 9, 1–13. (doi:10.2174/1874453201609010001)

[7] Menchetti M, Mori E 2014 Worldwide impact of alien parrots (Aves Psittaciformes) on native biodiversity and environment: a review. Ethol. Ecol. Evol. 26, 172–194. (doi:10.1080/03949370.2014.905981)

[8] Menchetti M, Mori E, Angelici FM 2016 Effects of the recent world invasion by ring-necked parakeets *Psittacula krameri*. In Problematic wildlife (ed. FM Angelici), pp. 253–266. Berlin, Germany: Springer International Publishing.

[9] Carboneras C et al. 2017 A prioritised list of invasive alien species to assist the effective implementation of EU legislation. J. Appl. Ecol. 55, 539–547. (doi:10.1111/1365-2664.12997)

[10] Turbé A et al. 2017 Assessing the assessments: evaluation of four impact assessment protocols for invasive alien species. Divers. Distrib. 23, 297–307. (doi:10.1111/ddi.12528)

[11] Peck HL, Pringle HE, Marshall HH, Owens IPF, Lord AM 2014 Experimental evidence of impacts of an invasive parakeet on foraging behavior of native birds. Behav. Ecol. 25, 582–590. (doi10.1093/beheco/aru025) 24822022 10.1093/beheco/aru025 PMC4014307

[12] Le Louarn M, Couillens B, Deschamps-Cottin M, Clergeau P 2016 Interference competition between an invasive parakeet and native bird species at feeding sites. J. Ethol. 34, 291–298. (doi:10.1007/s10164-016-0474-8) 27829702 10.1007/s10164-016-0474-8 PMC5080312

[13] Strubbe D, Matthysen E 2009 Experimental evidence for nest-site competition between invasive ring-necked parakeets (*Psittacula krameri*) and native nuthatches (*Sitta europaea*). Biol. Conserv. 142, 1588–1594. (doi:10.1016/j.biocon.2009.02.026)

[14] Orchan Y, Chiron F, Shwartz A, Kark S 2013 The complex interaction network among multiple invasive bird species in a cavity-nesting community. Biol. Invasions 15, 429–445. (doi:10.1007/s10530-012-0298-6)

[15] Dodaro G, Battisti C 2014 Rose-ringed parakeet (*Psittacula krameri*) and starling (*Sturnus vulgaris*) syntopics in a Mediterranean urban park: evidence for competition in nest-site selection? Belg. J. Zool. 144, 5–14.

[16] Hernández-Brito D, Carrete M, Popa-Lisseanu AG, Ibáñez C, Tella JL 2014 Crowding in the city: losing and winning competitors of an invasive bird. PLoS ONE 9, e100593 (doi:10.1371/journal.pone.0100593) 24945439 10.1371/journal.pone.0100593 PMC4063952

[17] Charter M, Izhaki I, Mocha YB, Kark S 2016 Nest-site competition between invasive and native cavity nesting birds and its implication for conservation. J. Environ. Manage. 181, 129–134. (doi:10.1016/j.jenvman.2016.06.021) 27341373 10.1016/j.jenvman.2016.06.021

[18] Yosef R, Zduniak P, Żmihorski M 2016 Invasive ring-necked parakeet negatively affects indigenous Eurasian hoopoe. Ann. Zool. Fennici 53, 281–287. (doi:10.5735/086.053.0605)

[19] Mori E, Ancillotto L, Menchetti M, Strubbe D 2017 ‘The early bird catches the nest’: possible competition between scops owls and ring-necked parakeets. Anim. Conserv. 20, 463–470. (doi:10.1111/acv.12334)

[20] Alcalde J, Juste J, Paunović M 2016 *Nyctalus lasiopterus*. The IUCN Red List of Threatened Species 2016: see http://www.iucnredlist.org/details/14918/0. Downloaded on 27 November 2017.

[21] Ibáñez C, Guillén A, Aguirre-Mendi PT, Juste J, Schreur G, Cordero AI, Popa-Lisseanu AG 2009 Sexual segregation in Iberian noctule bats. J. Mammal. 90, 235–243. (doi:10.1644/08-MAMM-A-037.1)

[22] Popa-Lisseanu AG, Bontadina F, Mora O, Ibáñez C 2008 Highly structured fission–fusion societies in an aerial hawking, carnivorous bat. Anim. Behav. 75, 471–482. (doi:10.1016/j.anbehav.2007.05.011)

[23] Fortuna MA, Popa-Lisseanu AG, Ibáñez C, Bascompte J 2009 The roosting spatial network of a bird-predator bat. Ecology 90, 934–944. (doi:10.1890/08-0174.1) 19449689 10.1890/08-0174.1

[24] Popa-Lisseanu AG, Bontadina F, Ibáñez C 2009 Giant noctule bats face conflicting constraints between roosting and foraging in a fragmented and heterogeneous landscape. J. zool. 278, 126–133. (doi:10.1111/j.1469-7998.2009.00556.x)

[25] Papadatou E et al. 2012 Comparing survival among species with imperfect detection using multilevel analysis of mark—recapture data: a case study on bats. Ecography 35, 153–161. (doi:10.1111/j.1600-0587.2011.07084.x)

[26] Santos JD, Meyer CF, Ibáñez C, Popa-Lisseanu AG, Juste J 2016 Dispersal and group formation dynamics in a rare and endangered temperate forest bat (*Nyctalus lasiopterus*, *Chiroptera: Vespertilionidae*). Ecol. Evol. 6, 8193–8204. (doi:10.1002/ece3.2330) 27878088 10.1002/ece3.2330 PMC5108270

[27] Cardador L, Carrete M, Gallardo B, Tella JL 2016 Combining trade data and niche modelling improves predictions of the origin and distribution of non-native European populations of a globally invasive species. J. Biogeogr. 43, 967–978. (doi:10.1111/jbi.12694)

[28] Abellán P, Tella JL, Carrete M, Cardador L, Anadón JD. 2017 Climatic matching drives spread rate but not establishment success in recent unintentional bird introductions. Proc. Natl Acad. Sci. USA 114, 9385–9390. (doi:10.1073/pnas.1704815114) 28784783 10.1073/pnas.1704815114 PMC5584426

[29] Hayes JP, Ober HK, Sherwin RE 2009 Survey and monitoring of bats. In Ecological and behavioral methods for the study of bats, 2nd edn (eds Kunz TH, Parsons S), pp 112–129. Baltimore, MD: The Johns Hopkins University Press.

[30] Kunz TH, Betke M, Hristov NI, Vonhof MJ 2009 Methods for assessing colony size, population size, and relative abundance of bats. In Ecological and behavioral methods for the study of bats, 2nd edn (eds Kunz TH, Parsons S), pp 133–157. Baltimore, MD: The Johns Hopkins University Press.

[31] Carrete M, Hernández-Brito D, Tella JL In preparation. Management of invasive parrot species: method and demographic feasibility.

[32] Moilanen A, Hanski I 1998 Metapopulation dynamics: effects of habitat quality and landscape structure. Ecology 79, 2503–2515. (doi:10.1890/0012-9658(1998)079[2503:MDEOHQ]2.0.CO;2)

[33] Burnham KP, Anderson DR 2002 Model selection and multimodel inference: a practical information-theoretic approach. New York, NY: Springer-Verlag.

[34] R Core Team. 2013 R: a language and environment for statistical computing. Vienna, Austria: R Foundation for Statistical Computing See http://www.R-project.org/.

[35] Sol D, González-Lagos C, Lapiedra O, Díaz M 2017 Why are exotic birds so successful in urbanized environments? In Ecology and conservation of birds in urban environments (eds E Murgui, M Hedblom), pp. 75–89. Berlin, Germany: Springer International Publishing.

[36] Ibáñez C, Juste J, García-Mudarra JL, Agirre-Mendi PT 2001 Bat predation on nocturnally migrating birds. Proc. Natl Acad. Sci. USA 98, 9700–9702. (doi:10.1073/pnas.171140598) 11493689 10.1073/pnas.171140598 PMC55515

[37] Ibáñez C, Popa-Lisseanu AG, Pastor-Beviá D, García-Mudarra J, Juste J 2016 Concealed by darkness: interactions between predatory bats and nocturnally migrating songbirds illuminated by DNA sequencing. Mol. Ecol. 25, 5254–5263. (doi10.1111/mec.13831) 27575398 10.1111/mec.13831

[38] Covas L, Senar JC, Roqué L, Quesada J 2017 Records of fatal attacks by rose-ringed parakeets *Psittacula krameri* on native avifauna. Rev. Catalana Ornitol. 33, 45–49.

[39] Menchetti M, Scalera R, Mori E 2014 First record of a possibly overlooked impact by alien parrots on a bat (*Nyctalus leisleri*). Hystrix 25, 61–62. (doi:10.4404/hystrix-25.1-9989)

[40] Hernández-Brito D, Luna A, Carrete M, Tella JL 2014 Alien rose-ringed parakeets (*Psittacula krameri*) attack black rats (*Rattus rattus*) sometimes resulting in death. Hystrix 25, 121–123. (doi:10.4404/hystrix-25.2-10992)

[41] Barclay RMR, Harder LD 2003 Life histories of bats: life in the slow lane. In Bat ecology (eds Kunz TH, Fenton MB), pp 209–253. Chicago, IL: Chicago University Press.

[42] Blackburn TM, Pettorelli N, Katzner T, Gompper ME, Mock K, Garner TWJ, Altwegg R, Redpath S, Gordon IJ 2010 Dying for conservation: eradicating invasive alien species in the face of opposition. Anim. Conserv. 13, 227–228. (doi:10.1111/j.1469-1795.2010.00372.x)

[43] Carrete M, Tella JL 2008 Non-native wildlife risk assessment: a call for scientific inquiry. Front. Ecol. Environ. 10, 466–467. (doi:10.1890/1540-9295-6.9.466.b)

[44] Dyer EE et al. 2017 The global distribution and drivers of alien bird species richness. PLoS Biol. 15, e2000942 (doi:10.1371/journal.pbio.2000942) 28081142 10.1371/journal.pbio.2000942 PMC5230740

[45] Ancillotto L, Tomassini A, Russo D 2015 The fancy city life: Kuhl's pipistrelle, *Pipistrellus kuhlii*, benefits from urbanization. Wildl. Res. 42, 598–606. (doi:10.1071/WR15003)

[46] Russo D, Ancillotto L 2015 Sensitivity of bats to urbanization: a review. Mamm. Biol. 80, 205–212. (doi:10.1016/j.mambio.2014.10.003) 10.1016/j.mambio.2014.10.003 PMC7094881 32226358

[47] Rydell J, Eklöf J, Sánchez-Navarro S 2017 Age of enlightenment: long-term effects of outdoor aesthetic lights on bats in churches. R. Soc. open sci. 4, 161077 (doi:10.1098/rsos.161077) 28878962 10.1098/rsos.161077 PMC5579077

[48] Haarsma AJ, van der Graaf C. 2013 Halsbandparkieten, een bredeiging voor Rosse vleermuizen? Levende Nat. 114, 10–13.

[49] Davidson AD, Hewitt CL 2014 How often are invasion-induced ecological impacts missed? Biol. Invasions 16, 1165–1173. (doi:10.1007/s10530-013-0570-4)

[50] Welc JN, Leppanen C 2017 The threat of invasive species to bats: a review. Mammal Rev. 47, 277–290. (doi:10.1111/mam.12099)

