# Supplementary material for: Nest-site competition and killing by invasive parakeets cause the decline of a threatened bat population
Source: R Soc Open Sci. 2018 May 9;5(5):172477. doi: 10.1098/rsos.172477 (PMC5990744; doi:10.1098/rsos.172477)
Supplement: Explanatory variables for occupation of cavities by noctules [file rsos172477supp1.docx]

| cavity | occupied | size | height (in m) | nndk (in m) | agregk | nndn (in m) | agregn | year |
| --- | --- | --- | --- | --- | --- | --- | --- | --- |
| 1 | 0 | 3 | 5.4 | 6.17 | 129.05 | 96.34 | 41.47 | 2013 |
| 2 | 0 | 1 | 9 | 4.42 | 135.29 | 43.82 | 44.17 | 2013 |
| 3 | 0 | 2 | 12.6 | 45.19 | 133.62 | 12.58 | 44.03 | 2013 |
| 4 | 1 | 2 | 9 | 33.44 | 134.54 | 70.48 | 43.30 | 2013 |
| 6 | 0 | 3 | 9.9 | 17.67 | 136.78 | 34.18 | 45.75 | 2013 |
| 7 | 0 | 3 | 18 | 17.67 | 136.78 | 34.18 | 45.75 | 2013 |
| 9 | 0 | 2 | 5.4 | 8.63 | 113.69 | 22.61 | 37.75 | 2013 |
| 10 | 0 | 3 | 7 | 16.94 | 114.50 | 28.84 | 38.08 | 2013 |
| 11 | 0 | 2 | 12.6 | 16.94 | 114.50 | 28.84 | 38.08 | 2013 |
| 13 | 0 | 1 | 14.4 | 9.23 | 136.25 | 26.51 | 45.08 | 2013 |
| 14 | 0 | 3 | 4.5 | 9.23 | 136.25 | 26.51 | 45.08 | 2013 |
| 17 | 0 | 3 | 7.2 | 13.06 | 120.88 | 77.59 | 40.03 | 2013 |
| 18 | 0 | 1 | 7.2 | 21.96 | 118.89 | 64.10 | 39.68 | 2013 |
| 19 | 0 | 2 | 10.8 | 21.96 | 118.89 | 64.10 | 39.68 | 2013 |
| 20 | 0 | 2 | 3.6 | 81.00 | 117.39 | 64.37 | 40.07 | 2013 |
| 21 | 0 | 2 | 14.4 | 81.00 | 117.39 | 64.37 | 40.07 | 2013 |
| 22 | 0 | 2 | 5.4 | 78.08 | 119.92 | 37.75 | 41.09 | 2013 |
| 23 | 0 | 2 | 7.2 | 70.28 | 121.16 | 26.61 | 41.44 | 2013 |
| 24 | 0 | 2 | 7.2 | 70.28 | 121.16 | 26.61 | 41.44 | 2013 |
| 25 | 0 | 3 | 13.5 | 36.44 | 122.45 | 29.88 | 41.29 | 2013 |
| 26 | 0 | 3 | 19.8 | 36.44 | 122.45 | 29.88 | 41.29 | 2013 |
| 27 | 0 | 2 | 6.3 | 40.37 | 126.83 | 31.15 | 42.66 | 2013 |
| 28 | 0 | 1 | 6.3 | 40.37 | 126.83 | 31.15 | 42.66 | 2013 |
| 29 | 0 | 1 | 9 | 7.41 | 136.47 | 24.70 | 45.34 | 2013 |
| 30 | 0 | 2 | 30.6 | 7.41 | 136.47 | 24.70 | 45.34 | 2013 |
| 32 | 0 | 1 | 6.5 | 39.20 | 126.99 | 30.98 | 42.72 | 2013 |
| 33 | 0 | 2 | 9 | 38.33 | 129.12 | 37.79 | 44.12 | 2013 |
| 34 | 0 | 1 | 9 | 38.33 | 129.12 | 37.79 | 44.12 | 2013 |
| 35 | 1 | 2 | 12.6 | 65.73 | 125.87 | 22.84 | 41.93 | 2013 |
| 36 | 0 | 1 | 4.5 | 65.73 | 125.87 | 8.10 | 41.94 | 2013 |
| 37 | 0 | 2 | 10.8 | 68.44 | 125.50 | 3.99 | 42.80 | 2013 |
| 38 | 0 | 1 | 4.5 | 72.11 | 125.51 | 7.36 | 42.93 | 2013 |
| 39 | 0 | 1 | 4.5 | 54.23 | 127.27 | 16.74 | 43.47 | 2013 |
| 40 | 0 | 2 | 9 | 50.33 | 123.89 | 19.17 | 41.91 | 2013 |
| 41 | 0 | 2 | 15.3 | 50.33 | 123.89 | 19.17 | 41.91 | 2013 |
| 42 | 0 | 1 | 6.3 | 50.33 | 123.89 | 19.17 | 41.91 | 2013 |
| 43 | 0 | 3 | 8.1 | 79.72 | 127.27 | 26.65 | 44.30 | 2013 |
| 45 | 0 | 1 | 12.6 | 54.58 | 130.13 | 58.81 | 45.00 | 2013 |
| 46 | 0 | 1 | 9 | 54.58 | 130.13 | 58.81 | 45.00 | 2013 |
| 47 | 0 | 1 | 10.8 | 38.34 | 129.91 | 50.23 | 44.54 | 2013 |
| 48 | 0 | 2 | 5.4 | 111.14 | 121.64 | 39.94 | 42.88 | 2013 |
| 49 | 0 | 1 | 5.4 | 111.14 | 121.64 | 39.94 | 42.88 | 2013 |
| 50 | 0 | 1 | 5.4 | 79.47 | 123.94 | 47.27 | 43.97 | 2013 |
| 51 | 0 | 1 | 5.4 | 91.05 | 123.72 | 29.51 | 43.61 | 2013 |
| 52 | 0 | 1 | 9 | 91.05 | 123.72 | 29.51 | 43.61 | 2013 |
| 53 | 0 | 1 | 7.2 | 101.01 | 123.21 | 23.64 | 43.31 | 2013 |
| 54 | 0 | 2 | 9 | 37.79 | 128.16 | 63.44 | 45.42 | 2013 |
| 55 | 0 | 1 | 9.9 | 58.37 | 124.52 | 93.78 | 44.93 | 2013 |
| 57 | 0 | 1 | 10.8 | 55.64 | 122.50 | 107.49 | 44.79 | 2013 |
| 58 | 0 | 2 | 15.3 | 7.56 | 137.37 | 4.42 | 45.68 | 2013 |
| 59 | 0 | 3 | 9.9 | 7.56 | 137.37 | 4.42 | 45.68 | 2013 |
| 60 | 0 | 1 | 6.3 | 44.80 | 123.31 | 61.32 | 45.86 | 2013 |
| 61 | 0 | 1 | 10.8 | 47.27 | 122.94 | 65.28 | 45.69 | 2013 |
| 62 | 0 | 1 | 12.6 | 53.53 | 122.40 | 62.58 | 45.61 | 2013 |
| 63 | 0 | 1 | 10.8 | 56.54 | 121.88 | 58.80 | 45.57 | 2013 |
| 65 | 0 | 2 | 12.6 | 38.29 | 122.92 | 25.17 | 46.51 | 2013 |
| 67 | 0 | 2 | 18.8 | 43.38 | 122.84 | 11.93 | 46.90 | 2013 |
| 68 | 0 | 1 | 10.8 | 48.39 | 122.75 | 13.92 | 47.05 | 2013 |
| 69 | 0 | 1 | 13.5 | 48.39 | 122.75 | 13.92 | 47.05 | 2013 |
| 70 | 0 | 2 | 8.1 | 34.60 | 124.05 | 3.60 | 46.32 | 2013 |
| 71 | 1 | 2 | 11.7 | 34.60 | 124.05 | 80.86 | 46.31 | 2013 |
| 72 | 0 | 3 | 11.7 | 34.60 | 124.05 | 1.00 | 46.31 | 2013 |
| 73 | 0 | 2 | 12.6 | 28.53 | 137.89 | 3.60 | 45.46 | 2013 |
| 74 | 0 | 1 | 7.2 | 28.53 | 137.89 | 9.00 | 45.46 | 2013 |
| 75 | 0 | 1 | 9.9 | 28.53 | 137.89 | 6.30 | 45.46 | 2013 |
| 76 | 0 | 1 | 10.3 | 28.53 | 137.89 | 5.90 | 45.46 | 2013 |
| 78 | 1 | 2 | 16.2 | 28.53 | 137.89 | 28.04 | 45.46 | 2013 |
| 79 | 0 | 1 | 9.9 | 58.91 | 120.74 | 39.30 | 45.85 | 2013 |
| 80 | 0 | 2 | 9.9 | 58.91 | 120.74 | 39.30 | 45.85 | 2013 |
| 81 | 0 | 2 | 4.5 | 52.08 | 121.50 | 38.81 | 45.94 | 2013 |
| 83 | 0 | 1 | 17.1 | 52.08 | 121.50 | 38.81 | 45.94 | 2013 |
| 84 | 0 | 2 | 12.6 | 52.08 | 121.50 | 38.81 | 45.94 | 2013 |
| 85 | 0 | 3 | 13.5 | 52.08 | 121.50 | 38.81 | 45.94 | 2013 |
| 86 | 0 | 1 | 9 | 52.78 | 121.60 | 45.50 | 45.80 | 2013 |
| 87 | 0 | 2 | 14.4 | 52.78 | 121.60 | 45.50 | 45.80 | 2013 |
| 88 | 0 | 2 | 14.9 | 52.78 | 121.60 | 45.50 | 45.80 | 2013 |
| 89 | 0 | 3 | 14 | 52.78 | 121.60 | 45.50 | 45.80 | 2013 |
| 90 | 0 | 1 | 8.1 | 18.88 | 125.90 | 42.74 | 47.00 | 2013 |
| 91 | 0 | 2 | 9.9 | 18.88 | 125.90 | 42.74 | 47.00 | 2013 |
| 92 | 0 | 2 | 16.2 | 23.67 | 128.36 | 83.93 | 46.55 | 2013 |
| 93 | 0 | 3 | 10.8 | 23.67 | 128.36 | 83.93 | 46.55 | 2013 |
| 94 | 0 | 3 | 10 | 23.67 | 128.36 | 83.93 | 46.55 | 2013 |
| 95 | 0 | 3 | 9 | 23.67 | 128.36 | 83.93 | 46.55 | 2013 |
| 96 | 0 | 2 | 18 | 11.84 | 127.99 | 80.94 | 46.67 | 2013 |
| 97 | 0 | 2 | 21.6 | 11.84 | 127.99 | 80.94 | 46.67 | 2013 |
| 98 | 0 | 1 | 11.7 | 16.57 | 129.18 | 87.71 | 46.13 | 2013 |
| 99 | 0 | 2 | 8.1 | 16.57 | 129.18 | 87.71 | 46.13 | 2013 |
| 100 | 0 | 2 | 8.5 | 16.57 | 129.18 | 87.71 | 46.13 | 2013 |
| 101 | 0 | 2 | 20.7 | 16.20 | 131.93 | 55.45 | 47.15 | 2013 |
| 102 | 0 | 2 | 18 | 21.67 | 127.27 | 58.06 | 47.15 | 2013 |
| 103 | 0 | 2 | 13.5 | 12.70 | 130.84 | 32.87 | 48.89 | 2013 |
| 104 | 1 | 2 | 18 | 7.41 | 137.51 | 28.04 | 44.79 | 2013 |
| 105 | 0 | 2 | 18 | 14.92 | 130.52 | 33.64 | 48.96 | 2013 |
| 106 | 0 | 3 | 10.8 | 40.08 | 127.64 | 39.40 | 48.72 | 2013 |
| 107 | 0 | 2 | 14.4 | 40.08 | 127.64 | 39.40 | 48.72 | 2013 |
| 108 | 0 | 3 | 16.2 | 39.36 | 128.17 | 33.24 | 48.91 | 2013 |
| 109 | 0 | 3 | 7.2 | 39.36 | 128.17 | 33.24 | 48.91 | 2013 |
| 110 | 0 | 1 | 12.6 | 6.18 | 133.57 | 17.52 | 50.74 | 2013 |
| 111 | 0 | 1 | 13 | 6.18 | 133.57 | 17.52 | 50.74 | 2013 |
| 112 | 0 | 2 | 16.2 | 6.18 | 133.57 | 17.52 | 50.74 | 2013 |
| 114 | 0 | 1 | 18 | 3.99 | 133.52 | 20.75 | 50.81 | 2013 |
| 115 | 0 | 2 | 18 | 3.99 | 133.52 | 20.75 | 50.81 | 2013 |
| 116 | 0 | 3 | 12.6 | 2.37 | 133.66 | 19.65 | 50.81 | 2013 |
| 117 | 0 | 2 | 15.3 | 2.37 | 133.66 | 19.65 | 50.81 | 2013 |
| 119 | 1 | 2 | 12.6 | 9.03 | 135.58 | 9.03 | 49.55 | 2013 |
| 120 | 0 | 3 | 17.1 | 19.05 | 136.62 | 26.98 | 49.67 | 2013 |
| 121 | 0 | 2 | 13.5 | 19.05 | 136.62 | 26.98 | 49.67 | 2013 |
| 122 | 0 | 3 | 14 | 19.05 | 136.62 | 26.98 | 49.67 | 2013 |
| 123 | 0 | 3 | 6.3 | 11.97 | 137.13 | 28.76 | 49.81 | 2013 |
| 125 | 0 | 1 | 5.4 | 16.57 | 136.79 | 34.08 | 49.48 | 2013 |
| 126 | 0 | 3 | 16.2 | 16.57 | 136.79 | 34.08 | 49.48 | 2013 |
| 127 | 0 | 2 | 9 | 60.84 | 131.45 | 84.27 | 43.51 | 2013 |
| 128 | 0 | 3 | 16.2 | 7.41 | 137.23 | 31.71 | 49.65 | 2013 |
| 129 | 0 | 2 | 5.4 | 5.75 | 137.29 | 31.35 | 49.58 | 2013 |
| 130 | 0 | 3 | 23.5 | 5.75 | 137.29 | 31.35 | 49.58 | 2013 |
| 131 | 0 | 2 | 21.6 | 5.77 | 137.71 | 35.54 | 48.95 | 2013 |
| 133 | 0 | 1 | 7.2 | 17.69 | 138.22 | 29.98 | 48.03 | 2013 |
| 134 | 0 | 2 | 10.8 | 17.69 | 138.22 | 29.98 | 48.03 | 2013 |
| 135 | 0 | 2 | 11.7 | 58.19 | 124.79 | 64.86 | 49.37 | 2013 |
| 136 | 0 | 1 | 12.6 | 58.19 | 124.79 | 64.86 | 49.37 | 2013 |
| 137 | 0 | 3 | 7.2 | 58.19 | 124.79 | 64.86 | 49.37 | 2013 |
| 139 | 0 | 3 | 19.8 | 58.19 | 124.79 | 64.86 | 49.37 | 2013 |
| 140 | 0 | 3 | 9 | 26.67 | 123.34 | 33.39 | 47.75 | 2013 |
| 141 | 0 | 2 | 15.3 | 40.77 | 121.06 | 6.30 | 48.52 | 2013 |
| 142 | 1 | 1 | 21.6 | 40.77 | 121.06 | 31.32 | 48.51 | 2013 |
| 145 | 0 | 2 | 18 | 12.70 | 122.03 | 63.29 | 47.91 | 2013 |
| 148 | 0 | 3 | 15.3 | 63.29 | 119.13 | 54.26 | 47.99 | 2013 |
| 149 | 0 | 2 | 6.3 | 44.97 | 133.61 | 72.71 | 45.01 | 2013 |
| 150 | 0 | 3 | 15.3 | 44.97 | 133.61 | 72.71 | 45.01 | 2013 |
| 151 | 0 | 3 | 7.2 | 59.29 | 114.81 | 51.38 | 48.30 | 2013 |
| 152 | 0 | 3 | 8 | 59.29 | 114.81 | 51.38 | 48.30 | 2013 |
| 153 | 0 | 2 | 16.2 | 59.29 | 114.81 | 51.38 | 48.30 | 2013 |
| 154 | 0 | 2 | 14.4 | 59.29 | 114.81 | 51.38 | 48.30 | 2013 |
| 155 | 0 | 3 | 10.8 | 51.78 | 115.51 | 50.62 | 48.49 | 2013 |
| 156 | 0 | 3 | 14.4 | 44.50 | 116.23 | 50.62 | 48.64 | 2013 |
| 157 | 0 | 2 | 6.3 | 44.58 | 117.00 | 27.53 | 49.21 | 2013 |
| 158 | 1 | 2 | 9.9 | 31.64 | 120.05 | 4.50 | 48.32 | 2013 |
| 160 | 0 | 2 | 9 | 31.64 | 120.05 | 0.90 | 48.33 | 2013 |
| 161 | 0 | 2 | 9.9 | 31.64 | 120.05 | 1.00 | 48.33 | 2013 |
| 162 | 1 | 2 | 14.4 | 31.64 | 120.05 | 4.50 | 48.32 | 2013 |
| 163 | 0 | 1 | 15 | 31.64 | 120.05 | 0.60 | 48.33 | 2013 |
| 164 | 0 | 2 | 14.4 | 31.64 | 120.05 | 1.00 | 48.33 | 2013 |
| 165 | 0 | 2 | 13.5 | 29.98 | 120.62 | 0.30 | 47.53 | 2013 |
| 166 | 1 | 2 | 13.8 | 29.98 | 120.62 | 4.20 | 47.53 | 2013 |
| 167 | 1 | 2 | 18 | 29.98 | 120.62 | 4.20 | 47.52 | 2013 |
| 168 | 1 | 2 | 27 | 29.98 | 120.62 | 7.59 | 47.53 | 2013 |
| 169 | 0 | 2 | 29.5 | 29.98 | 120.62 | 2.50 | 47.54 | 2013 |
| 170 | 0 | 3 | 9.9 | 25.66 | 121.36 | 7.10 | 50.66 | 2013 |
| 171 | 0 | 3 | 12.6 | 6.96 | 122.99 | 2.40 | 49.84 | 2013 |
| 172 | 1 | 2 | 15 | 6.96 | 122.99 | 25.99 | 49.84 | 2013 |
| 173 | 1 | 1 | 17.1 | 34.75 | 122.16 | 13.86 | 49.81 | 2013 |
| 174 | 0 | 2 | 25.2 | 34.75 | 122.16 | 8.10 | 49.82 | 2013 |
| 175 | 0 | 1 | 30.6 | 34.75 | 122.16 | 13.50 | 49.82 | 2013 |
| 177 | 0 | 1 | 12.6 | 39.81 | 133.38 | 64.64 | 44.28 | 2013 |
| 178 | 0 | 2 | 12.6 | 39.81 | 133.38 | 64.64 | 44.28 | 2013 |
| 179 | 0 | 2 | 11.7 | 26.51 | 123.48 | 8.10 | 48.04 | 2013 |
| 180 | 1 | 2 | 19.8 | 26.51 | 123.48 | 2.20 | 48.01 | 2013 |
| 181 | 0 | 1 | 21.6 | 26.51 | 123.48 | 0.40 | 48.01 | 2013 |
| 182 | 1 | 3 | 22 | 26.51 | 123.48 | 2.20 | 48.01 | 2013 |
| 183 | 1 | 2 | 26.1 | 26.51 | 123.48 | 4.10 | 48.01 | 2013 |
| 185 | 0 | 2 | 10.8 | 22.17 | 123.84 | 4.79 | 51.02 | 2013 |
| 186 | 0 | 3 | 13.5 | 22.17 | 123.84 | 4.79 | 51.02 | 2013 |
| 188 | 0 | 3 | 9.9 | 37.62 | 122.59 | 15.74 | 49.98 | 2013 |
| 189 | 0 | 3 | 9.9 | 33.64 | 122.45 | 16.31 | 50.01 | 2013 |
| 190 | 0 | 1 | 31.5 | 44.28 | 128.83 | 36.30 | 50.10 | 2013 |
| 191 | 0 | 1 | 9 | 68.74 | 124.24 | 68.74 | 44.41 | 2013 |
| 192 | 0 | 3 | 14.4 | 28.49 | 128.57 | 41.57 | 51.25 | 2013 |
| 196 | 0 | 2 | 7.2 | 9.03 | 127.64 | 17.30 | 51.20 | 2013 |
| 197 | 0 | 2 | 8.1 | 9.03 | 127.64 | 17.30 | 51.20 | 2013 |
| 198 | 0 | 2 | 16.2 | 9.03 | 127.64 | 17.30 | 51.20 | 2013 |
| 199 | 0 | 2 | 9.9 | 9.03 | 127.64 | 17.30 | 51.20 | 2013 |
| 200 | 0 | 1 | 9.9 | 6.29 | 131.63 | 66.91 | 42.42 | 2013 |
| 201 | 0 | 2 | 14.4 | 6.29 | 131.63 | 66.91 | 42.42 | 2013 |
| 203 | 0 | 1 | 18 | 16.17 | 137.22 | 25.19 | 47.11 | 2013 |
| 205 | 0 | 2 | 21.6 | 17.82 | 128.35 | 17.69 | 51.19 | 2013 |
| 206 | 0 | 2 | 23.4 | 17.82 | 128.35 | 17.69 | 51.19 | 2013 |
| 207 | 0 | 1 | 6.3 | 21.21 | 128.86 | 17.26 | 51.06 | 2013 |
| 208 | 0 | 2 | 10 | 21.21 | 128.86 | 17.26 | 51.06 | 2013 |
| 209 | 0 | 2 | 16.2 | 21.21 | 128.86 | 17.26 | 51.06 | 2013 |
| 210 | 0 | 2 | 13.5 | 16.17 | 129.57 | 11.53 | 51.15 | 2013 |
| 211 | 0 | 2 | 14 | 16.17 | 129.57 | 11.53 | 51.15 | 2013 |
| 212 | 0 | 1 | 13.5 | 9.23 | 130.03 | 4.73 | 51.11 | 2013 |
| 213 | 0 | 1 | 14.4 | 9.23 | 130.03 | 4.73 | 51.11 | 2013 |
| 214 | 0 | 3 | 9 | 9.23 | 130.03 | 4.73 | 51.11 | 2013 |
| 215 | 0 | 2 | 10.8 | 13.03 | 129.92 | 8.24 | 51.16 | 2013 |
| 216 | 0 | 2 | 16.2 | 13.03 | 129.92 | 8.24 | 51.16 | 2013 |
| 217 | 0 | 2 | 23.4 | 13.03 | 129.92 | 8.24 | 51.16 | 2013 |
| 218 | 0 | 1 | 13.5 | 4.79 | 130.41 | 3.48 | 50.10 | 2013 |
| 219 | 1 | 2 | 21.6 | 4.79 | 130.41 | 3.48 | 50.09 | 2013 |
| 221 | 0 | 2 | 13.5 | 4.79 | 130.41 | 3.48 | 50.10 | 2013 |
| 223 | 0 | 1 | 10.8 | 7.59 | 130.64 | 4.74 | 51.13 | 2013 |
| 224 | 0 | 3 | 25.2 | 7.59 | 130.64 | 4.74 | 51.13 | 2013 |
| 226 | 1 | 1 | 10.8 | 40.50 | 131.92 | 38.81 | 50.28 | 2013 |
| 227 | 1 | 2 | 15.3 | 3.99 | 130.31 | 3.48 | 50.04 | 2013 |
| 228 | 0 | 1 | 12.6 | 23.30 | 136.71 | 26.04 | 46.93 | 2013 |
| 229 | 0 | 3 | 6.3 | 13.76 | 125.24 | 16.55 | 50.79 | 2013 |
| 230 | 0 | 2 | 23.4 | 13.76 | 125.24 | 16.55 | 50.79 | 2013 |
| 231 | 0 | 2 | 10.8 | 13.76 | 125.24 | 16.55 | 50.79 | 2013 |
| 232 | 0 | 3 | 13.5 | 5.77 | 124.01 | 22.40 | 50.83 | 2013 |
| 234 | 0 | 3 | 14.4 | 13.92 | 123.39 | 16.49 | 50.82 | 2013 |
| 236 | 0 | 3 | 13.5 | 28.20 | 122.33 | 12.35 | 50.76 | 2013 |
| 237 | 0 | 1 | 9 | 28.20 | 122.33 | 12.35 | 50.76 | 2013 |
| 238 | 0 | 2 | 21.6 | 28.20 | 122.33 | 12.35 | 50.76 | 2013 |
| 240 | 0 | 3 | 5.4 | 38.76 | 120.70 | 7.20 | 49.53 | 2013 |
| 241 | 0 | 2 | 23.4 | 38.76 | 120.70 | 9.23 | 49.53 | 2013 |
| 242 | 1 | 3 | 12.6 | 38.76 | 120.70 | 9.23 | 49.52 | 2013 |
| 243 | 0 | 3 | 7.2 | 41.72 | 119.82 | 9.03 | 49.31 | 2013 |
| 244 | 1 | 3 | 18 | 41.72 | 119.82 | 9.03 | 49.30 | 2013 |
| 245 | 0 | 1 | 25.2 | 41.72 | 119.82 | 7.20 | 49.31 | 2013 |
| 246 | 1 | 2 | 27 | 42.62 | 119.19 | 9.03 | 49.10 | 2013 |
| 247 | 0 | 3 | 9 | 42.62 | 119.19 | 9.03 | 49.12 | 2013 |
| 248 | 0 | 3 | 7.2 | 12.80 | 125.18 | 57.49 | 49.47 | 2013 |
| 249 | 0 | 3 | 19.8 | 12.80 | 125.18 | 57.49 | 49.47 | 2013 |
| 250 | 0 | 2 | 28.8 | 12.80 | 125.18 | 57.49 | 49.47 | 2013 |
| 251 | 0 | 3 | 10.8 | 12.80 | 125.18 | 57.49 | 49.47 | 2013 |
| 255 | 0 | 2 | 8.1 | 3.48 | 124.59 | 60.77 | 49.14 | 2013 |
| 258 | 0 | 2 | 9 | 14.84 | 124.48 | 55.68 | 49.49 | 2013 |
| 259 | 0 | 2 | 16.2 | 14.84 | 124.48 | 55.68 | 49.49 | 2013 |
| 263 | 0 | 2 | 10.8 | 35.78 | 136.05 | 40.50 | 46.38 | 2013 |
| 264 | 0 | 1 | 7.2 | 15.95 | 122.75 | 41.43 | 48.95 | 2013 |
| 265 | 0 | 2 | 12.6 | 15.95 | 122.75 | 41.43 | 48.95 | 2013 |
| 266 | 0 | 2 | 8.1 | 27.09 | 121.22 | 31.39 | 49.21 | 2013 |
| 268 | 0 | 2 | 12.6 | 15.47 | 124.45 | 31.46 | 50.23 | 2013 |
| 269 | 0 | 2 | 7.2 | 15.47 | 124.45 | 31.46 | 50.23 | 2013 |
| 270 | 0 | 1 | 9 | 19.50 | 120.98 | 34.75 | 49.30 | 2013 |
| 272 | 0 | 2 | 7 | 14.84 | 120.75 | 36.91 | 49.34 | 2013 |
| 273 | 0 | 2 | 8.1 | 69.34 | 114.40 | 46.26 | 48.30 | 2013 |
| 274 | 0 | 2 | 10.8 | 69.34 | 114.40 | 46.26 | 48.30 | 2013 |
| 275 | 0 | 2 | 14.4 | 69.34 | 114.40 | 46.26 | 48.30 | 2013 |
| 276 | 0 | 2 | 10.8 | 69.34 | 114.40 | 46.26 | 48.30 | 2013 |
| 277 | 0 | 2 | 9.9 | 124.35 | 108.62 | 56.81 | 46.12 | 2013 |
| 278 | 0 | 3 | 9 | 124.35 | 108.62 | 56.81 | 46.12 | 2013 |
| 279 | 0 | 3 | 12.6 | 129.42 | 108.46 | 61.39 | 46.07 | 2013 |
| 280 | 0 | 3 | 12.6 | 65.92 | 113.20 | 16.55 | 47.50 | 2013 |
| 281 | 0 | 2 | 10.8 | 65.92 | 113.20 | 16.55 | 47.50 | 2013 |
| 283 | 0 | 3 | 9 | 42.75 | 135.31 | 39.69 | 46.39 | 2013 |
| 284 | 0 | 1 | 7.2 | 62.01 | 113.97 | 9.00 | 47.05 | 2013 |
| 285 | 0 | 2 | 9.9 | 62.01 | 113.97 | 6.30 | 47.04 | 2013 |
| 286 | 1 | 2 | 16.2 | 62.01 | 113.97 | 12.35 | 47.04 | 2013 |
| 289 | 1 | 2 | 26.1 | 68.08 | 114.33 | 22.60 | 47.31 | 2013 |
| 290 | 0 | 1 | 27 | 83.29 | 113.51 | 17.77 | 48.06 | 2013 |
| 293 | 0 | 2 | 10.8 | 105.55 | 110.17 | 39.40 | 46.71 | 2013 |
| 294 | 0 | 2 | 16.2 | 97.03 | 111.55 | 28.99 | 47.29 | 2013 |
| 296 | 0 | 2 | 27 | 97.03 | 111.55 | 28.99 | 47.29 | 2013 |
| 297 | 0 | 1 | 13.5 | 116.09 | 110.84 | 49.99 | 47.04 | 2013 |
| 299 | 0 | 1 | 12.6 | 118.84 | 110.64 | 54.18 | 46.96 | 2013 |
| 300 | 0 | 3 | 19.8 | 118.84 | 110.64 | 54.18 | 46.96 | 2013 |
| 301 | 0 | 1 | 4 | 66.30 | 116.59 | 26.11 | 49.21 | 2013 |
| 302 | 0 | 2 | 5.4 | 83.22 | 115.59 | 38.05 | 48.84 | 2013 |
| 304 | 0 | 1 | 4 | 78.00 | 116.33 | 36.69 | 49.10 | 2013 |
| 305 | 0 | 1 | 5.4 | 68.51 | 116.10 | 25.89 | 48.98 | 2013 |
| 306 | 0 | 2 | 7.2 | 68.51 | 116.10 | 25.89 | 48.98 | 2013 |
| 307 | 0 | 1 | 4 | 74.59 | 115.36 | 24.30 | 48.74 | 2013 |
| 308 | 0 | 1 | 5.4 | 69.90 | 114.96 | 12.80 | 48.57 | 2013 |
| 309 | 0 | 2 | 7.2 | 69.90 | 114.96 | 12.80 | 48.57 | 2013 |
| 310 | 0 | 1 | 4 | 65.90 | 114.99 | 7.98 | 48.55 | 2013 |
| 311 | 0 | 2 | 5.4 | 65.90 | 114.99 | 7.98 | 48.55 | 2013 |
| 312 | 0 | 1 | 8.1 | 63.01 | 114.16 | 8.24 | 48.16 | 2013 |
| 313 | 0 | 2 | 8.1 | 63.01 | 114.16 | 8.24 | 48.16 | 2013 |
| 315 | 1 | 2 | 25.2 | 53.58 | 114.63 | 1.00 | 45.18 | 2013 |
| 316 | 1 | 3 | 25.2 | 53.58 | 114.63 | 1.00 | 45.18 | 2013 |
| 317 | 0 | 2 | 9.9 | 53.58 | 114.63 | 8.10 | 45.21 | 2013 |
| 318 | 1 | 2 | 18 | 53.58 | 114.63 | 7.20 | 45.18 | 2013 |
| 321 | 1 | 2 | 20.7 | 41.72 | 115.83 | 13.86 | 47.41 | 2013 |
| 324 | 0 | 2 | 23.4 | 27.95 | 117.33 | 14.25 | 48.76 | 2013 |
| 327 | 0 | 2 | 12.6 | 66.69 | 133.33 | 60.64 | 45.75 | 2013 |
| 328 | 0 | 2 | 7 | 66.69 | 133.33 | 60.64 | 45.75 | 2013 |
| 329 | 0 | 2 | 12.6 | 66.69 | 133.33 | 60.64 | 45.75 | 2013 |
| 331 | 0 | 3 | 5.4 | 26.67 | 117.69 | 19.45 | 49.04 | 2013 |
| 332 | 0 | 2 | 23.4 | 26.67 | 117.69 | 19.45 | 49.04 | 2013 |
| 333 | 0 | 3 | 16.2 | 19.65 | 118.83 | 30.55 | 49.38 | 2013 |
| 334 | 0 | 2 | 18 | 19.65 | 118.83 | 30.55 | 49.38 | 2013 |
| 337 | 0 | 2 | 27 | 26.21 | 135.75 | 35.63 | 50.76 | 2013 |
| 339 | 0 | 1 | 16.2 | 26.98 | 135.67 | 39.81 | 50.76 | 2013 |
| 340 | 0 | 1 | 16.2 | 36.54 | 135.37 | 25.62 | 50.68 | 2013 |
| 341 | 0 | 1 | 19.8 | 36.54 | 135.37 | 25.62 | 50.68 | 2013 |
| 342 | 0 | 2 | 18 | 36.54 | 135.37 | 25.62 | 50.68 | 2013 |
| 343 | 0 | 1 | 12.6 | 14.99 | 136.48 | 22.78 | 50.50 | 2013 |
| 344 | 0 | 3 | 15.3 | 8.10 | 135.73 | 8.24 | 50.62 | 2013 |
| 345 | 0 | 2 | 17.1 | 8.10 | 135.73 | 8.24 | 50.62 | 2013 |
| 346 | 0 | 2 | 12.6 | 8.10 | 135.73 | 8.24 | 50.62 | 2013 |
| 347 | 0 | 2 | 6.3 | 34.67 | 135.69 | 28.53 | 46.83 | 2013 |
| 348 | 0 | 2 | 8.1 | 34.67 | 135.69 | 28.53 | 46.83 | 2013 |
| 349 | 0 | 3 | 6.5 | 34.67 | 135.69 | 28.53 | 46.83 | 2013 |
| 351 | 0 | 1 | 9 | 17.30 | 137.99 | 9.23 | 49.60 | 2013 |
| 352 | 0 | 2 | 15.3 | 7.36 | 137.81 | 24.70 | 49.29 | 2013 |
| 353 | 0 | 3 | 7.2 | 7.36 | 137.81 | 24.70 | 49.29 | 2013 |
| 355 | 0 | 1 | 11.7 | 13.70 | 138.33 | 27.68 | 48.85 | 2013 |
| 357 | 0 | 3 | 9 | 31.35 | 138.45 | 43.15 | 48.38 | 2013 |
| 358 | 0 | 3 | 18 | 22.22 | 138.30 | 14.90 | 49.33 | 2013 |
| 359 | 0 | 2 | 10.8 | 22.22 | 138.30 | 14.90 | 49.33 | 2013 |
| 360 | 0 | 3 | 20.7 | 22.22 | 138.30 | 14.90 | 49.33 | 2013 |
| 361 | 0 | 3 | 18.5 | 22.22 | 138.30 | 14.90 | 49.33 | 2013 |
| 362 | 0 | 2 | 17.4 | 22.22 | 138.30 | 14.90 | 49.33 | 2013 |
| 363 | 0 | 3 | 16.2 | 22.22 | 138.30 | 14.90 | 49.33 | 2013 |
| 365 | 0 | 3 | 15.3 | 19.23 | 138.23 | 9.23 | 49.38 | 2013 |
| 366 | 0 | 3 | 13.5 | 19.23 | 138.23 | 9.23 | 49.38 | 2013 |
| 367 | 0 | 2 | 8.1 | 8.10 | 137.61 | 10.98 | 49.95 | 2013 |
| 368 | 0 | 2 | 7.2 | 8.10 | 137.61 | 10.98 | 49.95 | 2013 |
| 369 | 0 | 1 | 5.4 | 7.98 | 137.49 | 5.40 | 48.80 | 2013 |
| 370 | 1 | 2 | 10.8 | 7.98 | 137.49 | 35.39 | 48.79 | 2013 |
| 371 | 1 | 3 | 7.2 | 26.32 | 132.31 | 13.06 | 49.84 | 2013 |
| 373 | 1 | 3 | 14.4 | 37.08 | 133.46 | 13.92 | 49.85 | 2013 |
| 374 | 0 | 2 | 12.6 | 43.03 | 134.13 | 45.04 | 46.99 | 2013 |
| 375 | 0 | 3 | 9 | 43.03 | 134.13 | 45.04 | 46.99 | 2013 |
| 376 | 1 | 2 | 9 | 39.03 | 132.81 | 13.06 | 49.68 | 2013 |
| 377 | 1 | 2 | 14.4 | 24.62 | 133.65 | 1.80 | 47.35 | 2013 |
| 378 | 1 | 2 | 16.2 | 24.62 | 133.65 | 1.80 | 47.35 | 2013 |
| 380 | 0 | 2 | 9 | 24.14 | 134.99 | 14.84 | 48.57 | 2013 |
| 381 | 0 | 2 | 11.7 | 42.87 | 135.21 | 15.12 | 48.10 | 2013 |
| 382 | 0 | 2 | 11.7 | 19.65 | 136.13 | 14.83 | 49.00 | 2013 |
| 386 | 0 | 2 | 15.3 | 41.57 | 137.96 | 33.75 | 48.90 | 2013 |
| 387 | 0 | 2 | 12.6 | 41.57 | 137.96 | 33.75 | 48.90 | 2013 |
| 388 | 0 | 2 | 9.9 | 48.63 | 138.00 | 40.84 | 48.70 | 2013 |
| 389 | 0 | 2 | 4.5 | 48.63 | 138.00 | 40.84 | 48.70 | 2013 |
| 390 | 0 | 2 | 13.5 | 39.44 | 134.92 | 43.35 | 47.92 | 2013 |
| 391 | 0 | 3 | 5.4 | 52.55 | 137.99 | 44.73 | 48.58 | 2013 |
| 392 | 0 | 2 | 7.2 | 65.95 | 137.77 | 46.56 | 47.64 | 2013 |
| 393 | 0 | 2 | 6.3 | 61.17 | 137.90 | 43.38 | 47.63 | 2013 |
| 394 | 0 | 2 | 9.9 | 73.00 | 137.43 | 48.88 | 47.93 | 2013 |
| 395 | 0 | 2 | 15.3 | 73.00 | 137.43 | 48.88 | 47.93 | 2013 |
| 396 | 0 | 3 | 5.4 | 73.00 | 137.43 | 48.88 | 47.93 | 2013 |
| 397 | 0 | 1 | 7.2 | 73.00 | 137.43 | 48.88 | 47.93 | 2013 |
| 398 | 0 | 1 | 8.1 | 73.00 | 137.43 | 48.88 | 47.93 | 2013 |
| 399 | 0 | 2 | 16.2 | 74.08 | 137.34 | 48.00 | 47.89 | 2013 |
| 400 | 0 | 1 | 10.8 | 60.65 | 136.47 | 31.15 | 47.93 | 2013 |
| 401 | 0 | 2 | 12.6 | 60.65 | 136.47 | 31.15 | 47.93 | 2013 |
| 402 | 0 | 1 | 5.4 | 69.84 | 135.33 | 42.15 | 47.32 | 2013 |
| 403 | 0 | 1 | 5.4 | 38.69 | 133.47 | 45.10 | 47.17 | 2013 |
| 404 | 0 | 1 | 8.1 | 38.69 | 133.47 | 45.10 | 47.17 | 2013 |
| 405 | 0 | 3 | 8.1 | 42.87 | 133.32 | 52.91 | 46.89 | 2013 |
| 406 | 0 | 2 | 25.2 | 43.21 | 135.37 | 46.35 | 48.42 | 2013 |
| 407 | 0 | 2 | 10.8 | 43.21 | 135.37 | 46.35 | 48.42 | 2013 |
| 408 | 0 | 2 | 9.9 | 32.24 | 132.73 | 81.32 | 45.39 | 2013 |
| 409 | 0 | 3 | 12.6 | 32.24 | 132.73 | 81.32 | 45.39 | 2013 |
| 412 | 0 | 2 | 18.9 | 23.30 | 131.36 | 62.79 | 44.80 | 2013 |
| 414 | 0 | 2 | 12.6 | 58.82 | 131.56 | 75.93 | 45.60 | 2013 |
| 417 | 0 | 2 | 7.2 | 58.82 | 131.56 | 75.93 | 45.60 | 2013 |
| 419 | 0 | 3 | 5.4 | 41.61 | 132.49 | 64.64 | 46.31 | 2013 |
| 420 | 0 | 3 | 10 | 41.61 | 132.49 | 64.64 | 46.31 | 2013 |
| 421 | 0 | 2 | 18 | 41.61 | 132.49 | 64.64 | 46.31 | 2013 |
| 422 | 0 | 1 | 10.8 | 41.61 | 132.49 | 64.64 | 46.31 | 2013 |
| 423 | 0 | 1 | 8.1 | 9.72 | 130.63 | 84.94 | 42.05 | 2013 |
| 424 | 0 | 2 | 16.2 | 9.72 | 130.63 | 84.94 | 42.05 | 2013 |
| 425 | 0 | 2 | 7.2 | 17.13 | 137.09 | 3.99 | 47.90 | 2013 |
| 427 | 0 | 2 | 16.2 | 27.41 | 132.44 | 66.89 | 46.65 | 2013 |
| 428 | 0 | 2 | 6.3 | 27.41 | 132.44 | 66.89 | 46.65 | 2013 |
| 429 | 0 | 1 | 9 | 27.41 | 132.44 | 66.89 | 46.65 | 2013 |
| 430 | 0 | 2 | 11.7 | 27.41 | 132.44 | 66.89 | 46.65 | 2013 |
| 431 | 0 | 2 | 11.7 | 26.97 | 133.58 | 55.37 | 47.23 | 2013 |
| 432 | 0 | 2 | 18 | 26.97 | 133.58 | 55.37 | 47.23 | 2013 |
| 433 | 0 | 3 | 14.4 | 26.97 | 133.58 | 55.37 | 47.23 | 2013 |
| 434 | 0 | 3 | 14.4 | 18.59 | 132.86 | 64.84 | 47.09 | 2013 |
| 435 | 0 | 3 | 7.2 | 18.59 | 132.86 | 64.84 | 47.09 | 2013 |
| 436 | 0 | 2 | 16.2 | 12.80 | 131.71 | 78.57 | 46.78 | 2013 |
| 438 | 0 | 2 | 10.8 | 12.80 | 131.71 | 78.57 | 46.78 | 2013 |
| 439 | 0 | 2 | 9 | 4.79 | 131.67 | 81.68 | 46.99 | 2013 |
| 440 | 0 | 1 | 9 | 4.79 | 131.67 | 81.68 | 46.99 | 2013 |
| 441 | 0 | 2 | 18 | 4.79 | 131.67 | 81.68 | 46.99 | 2013 |
| 443 | 0 | 2 | 6.3 | 20.53 | 133.47 | 64.57 | 47.81 | 2013 |
| 444 | 0 | 1 | 6.3 | 22.14 | 132.25 | 56.73 | 48.17 | 2013 |
| 445 | 0 | 3 | 6.8 | 22.14 | 132.25 | 56.73 | 48.17 | 2013 |
| 446 | 0 | 2 | 7.2 | 14.25 | 137.12 | 7.20 | 46.80 | 2013 |
| 447 | 0 | 2 | 13 | 14.25 | 137.12 | 1.40 | 46.80 | 2013 |
| 448 | 1 | 3 | 14.4 | 14.25 | 137.12 | 67.91 | 46.80 | 2013 |
| 449 | 0 | 2 | 19.8 | 6.29 | 130.26 | 69.34 | 47.65 | 2013 |
| 450 | 0 | 2 | 12.6 | 31.48 | 132.10 | 38.09 | 48.92 | 2013 |
| 451 | 0 | 3 | 5.4 | 17.13 | 131.15 | 47.22 | 48.45 | 2013 |
| 452 | 0 | 3 | 6.3 | 27.98 | 131.24 | 34.25 | 48.86 | 2013 |
| 453 | 0 | 3 | 10.8 | 27.98 | 131.24 | 34.25 | 48.86 | 2013 |
| 454 | 0 | 3 | 5.4 | 18.94 | 136.26 | 26.61 | 49.53 | 2013 |
| 455 | 0 | 3 | 12.6 | 18.94 | 136.26 | 26.61 | 49.53 | 2013 |
| 456 | 0 | 1 | 13.5 | 33.79 | 127.85 | 41.01 | 49.65 | 2013 |
| 457 | 0 | 2 | 19.8 | 33.79 | 127.85 | 41.01 | 49.65 | 2013 |
| 458 | 0 | 2 | 23.4 | 33.79 | 127.85 | 41.01 | 49.65 | 2013 |
| 459 | 0 | 2 | 10.8 | 25.61 | 128.86 | 30.23 | 49.82 | 2013 |
| 460 | 0 | 2 | 14.4 | 24.36 | 129.38 | 25.66 | 49.96 | 2013 |
| 461 | 0 | 2 | 10.8 | 24.36 | 129.38 | 25.66 | 49.96 | 2013 |
| 462 | 0 | 2 | 8.1 | 24.36 | 129.38 | 25.66 | 49.96 | 2013 |
| 464 | 0 | 2 | 8.1 | 24.09 | 126.63 | 53.76 | 49.40 | 2013 |
| 465 | 0 | 2 | 12.6 | 1.48 | 124.25 | 56.83 | 49.11 | 2013 |
| 466 | 0 | 3 | 14.4 | 9.47 | 123.39 | 51.85 | 48.77 | 2013 |
| 467 | 0 | 1 | 9 | 38.48 | 135.67 | 42.65 | 48.47 | 2013 |
| 468 | 0 | 2 | 8.5 | 38.48 | 135.67 | 42.65 | 48.47 | 2013 |
| 469 | 0 | 2 | 11.7 | 38.48 | 135.67 | 42.65 | 48.47 | 2013 |
| 470 | 0 | 2 | 9.9 | 15.47 | 122.19 | 43.15 | 48.47 | 2013 |
| 471 | 0 | 2 | 9 | 7.56 | 119.36 | 16.17 | 48.09 | 2013 |
| 472 | 0 | 2 | 15.3 | 7.56 | 119.36 | 16.17 | 48.09 | 2013 |
| 473 | 1 | 2 | 14.4 | 14.84 | 118.45 | 0.40 | 45.20 | 2013 |
| 474 | 1 | 2 | 14 | 14.84 | 118.45 | 0.40 | 45.20 | 2013 |
| 475 | 1 | 3 | 15 | 14.84 | 118.45 | 0.60 | 45.20 | 2013 |
| 476 | 0 | 1 | 13.5 | 22.40 | 119.06 | 9.23 | 48.50 | 2013 |
| 477 | 0 | 2 | 14.4 | 22.40 | 119.06 | 9.23 | 48.50 | 2013 |
| 478 | 0 | 2 | 6.3 | 36.27 | 117.22 | 22.14 | 48.22 | 2013 |
| 479 | 0 | 2 | 9 | 36.27 | 117.22 | 22.14 | 48.22 | 2013 |
| 480 | 0 | 1 | 10.8 | 56.55 | 114.55 | 29.10 | 47.52 | 2013 |
| 481 | 0 | 2 | 9 | 48.74 | 115.27 | 17.81 | 47.93 | 2013 |
| 483 | 0 | 3 | 12.6 | 38.05 | 133.10 | 39.40 | 48.79 | 2013 |
| 484 | 1 | 3 | 9.9 | 20.60 | 122.76 | 8.63 | 49.39 | 2013 |
| 485 | 1 | 2 | 13.5 | 13.06 | 121.91 | 8.63 | 49.23 | 2013 |
| 486 | 1 | 3 | 13.5 | 30.59 | 135.80 | 51.09 | 47.57 | 2013 |
| 487 | 1 | 3 | 20.7 | 24.32 | 133.23 | 0.90 | 48.27 | 2013 |
| 488 | 1 | 3 | 21.6 | 24.32 | 133.23 | 0.90 | 48.27 | 2013 |
| 489 | 1 | 3 | 18 | 46.49 | 130.92 | 26.77 | 48.86 | 2013 |
| 491 | 0 | 3 | 9 | 16.20 | 131.31 | 1.80 | 48.89 | 2013 |
| 492 | 1 | 3 | 10.8 | 16.20 | 131.31 | 44.75 | 48.88 | 2013 |
| 493 | 1 | 3 | 15.3 | 81.54 | 123.57 | 2.70 | 40.46 | 2013 |
| 494 | 0 | 3 | 8.1 | 81.54 | 123.57 | 4.50 | 40.47 | 2013 |
| 495 | 1 | 3 | 12.6 | 81.54 | 123.57 | 2.70 | 40.46 | 2013 |
| 496 | 1 | 2 | 23.4 | 6.96 | 134.53 | 9.00 | 48.49 | 2013 |
| 497 | 1 | 2 | 14.4 | 6.96 | 134.53 | 9.00 | 48.49 | 2013 |
| 499 | 1 | 2 | 16.2 | 66.28 | 123.72 | 16.49 | 41.22 | 2013 |
| 500 | 1 | 3 | 13.5 | 100.08 | 124.74 | 51.17 | 42.56 | 2013 |
| 504 | 1 | 2 | 18 | 12.58 | 134.93 | 0.09 | 49.97 | 2013 |
| 517 | 0 | 2 | 9 | 31.15 | 126.14 | 127.22 | 40.58 | 2013 |
| 518 | 0 | 1 | 6.3 | 31.15 | 126.14 | 127.22 | 40.58 | 2013 |
| 520 | 0 | 1 | 14.4 | 23.64 | 126.41 | 121.56 | 40.62 | 2013 |
| 521 | 0 | 1 | 12.6 | 49.16 | 124.76 | 143.75 | 40.20 | 2013 |
| 522 | 0 | 3 | 9.9 | 49.16 | 124.76 | 143.75 | 40.20 | 2013 |
| 523 | 0 | 3 | 8.1 | 7.59 | 131.97 | 81.19 | 42.64 | 2013 |
| 524 | 0 | 2 | 17.1 | 7.59 | 131.97 | 81.19 | 42.64 | 2013 |
| 526 | 0 | 3 | 5 | 60.84 | 122.76 | 158.50 | 39.51 | 2013 |
| 527 | 0 | 3 | 9 | 60.84 | 122.76 | 158.50 | 39.51 | 2013 |
| 529 | 0 | 1 | 16.2 | 96.78 | 117.77 | 202.15 | 37.90 | 2013 |
| 530 | 0 | 2 | 9.9 | 83.14 | 112.62 | 232.22 | 36.04 | 2013 |
| 531 | 0 | 2 | 10.8 | 79.66 | 112.98 | 228.79 | 36.16 | 2013 |
| 532 | 0 | 2 | 18 | 75.32 | 113.53 | 223.99 | 36.34 | 2013 |
| 534 | 0 | 2 | 9.9 | 70.73 | 114.29 | 217.96 | 36.59 | 2013 |
| 536 | 0 | 2 | 9 | 19.15 | 119.99 | 163.89 | 38.44 | 2013 |
| 537 | 0 | 3 | 2.7 | 19.15 | 119.99 | 163.89 | 38.44 | 2013 |
| 538 | 0 | 1 | 10.8 | 27.50 | 124.19 | 123.66 | 39.85 | 2013 |
| 539 | 0 | 2 | 15.3 | 37.33 | 125.44 | 110.23 | 40.30 | 2013 |
| 540 | 0 | 2 | 19.8 | 9.03 | 132.06 | 82.56 | 42.70 | 2013 |
| 541 | 0 | 2 | 15.3 | 32.05 | 125.39 | 114.16 | 40.24 | 2013 |
| 542 | 0 | 2 | 10.8 | 19.45 | 127.27 | 96.86 | 40.89 | 2013 |
| 543 | 0 | 3 | 12.6 | 19.45 | 127.27 | 96.86 | 40.89 | 2013 |
| 544 | 0 | 2 | 12.6 | 33.75 | 128.34 | 79.55 | 41.39 | 2013 |
| 546 | 0 | 2 | 11.7 | 53.56 | 116.97 | 181.46 | 37.55 | 2013 |
| 547 | 0 | 3 | 12.6 | 29.51 | 138.12 | 18.59 | 46.90 | 2013 |
| 548 | 0 | 1 | 9.9 | 37.87 | 137.70 | 19.24 | 46.72 | 2013 |
| 549 | 0 | 2 | 8.1 | 13.70 | 132.97 | 71.64 | 43.11 | 2013 |
| 550 | 0 | 2 | 16.2 | 13.70 | 132.97 | 71.64 | 43.11 | 2013 |
| 551 | 0 | 2 | 14.4 | 30.59 | 137.70 | 13.03 | 46.50 | 2013 |
| 552 | 0 | 1 | 6.3 | 155.55 | 104.99 | 243.59 | 33.60 | 2013 |
| 553 | 0 | 3 | 5.4 | 160.65 | 104.45 | 248.47 | 33.42 | 2013 |
| 555 | 0 | 1 | 7.2 | 168.87 | 103.53 | 260.66 | 33.12 | 2013 |
| 556 | 0 | 1 | 7.2 | 175.81 | 102.83 | 265.90 | 32.89 | 2013 |
| 557 | 0 | 1 | 7.8 | 175.81 | 102.83 | 265.90 | 32.89 | 2013 |
| 558 | 0 | 1 | 9 | 189.45 | 101.44 | 279.16 | 32.44 | 2013 |
| 559 | 0 | 2 | 2.5 | 189.45 | 101.44 | 279.16 | 32.44 | 2013 |
| 560 | 0 | 3 | 3.5 | 189.45 | 101.44 | 279.16 | 32.44 | 2013 |
| 561 | 0 | 3 | 7.2 | 199.98 | 100.44 | 282.95 | 32.12 | 2013 |
| 562 | 0 | 2 | 7.2 | 199.98 | 100.44 | 282.95 | 32.12 | 2013 |
| 564 | 0 | 1 | 10.8 | 199.98 | 100.44 | 282.95 | 32.12 | 2013 |
| 567 | 0 | 2 | 13.5 | 16.20 | 132.72 | 61.97 | 42.86 | 2013 |
| 568 | 0 | 3 | 4.5 | 215.91 | 99.03 | 282.68 | 31.67 | 2013 |
| 569 | 0 | 2 | 4.5 | 215.91 | 99.03 | 282.68 | 31.67 | 2013 |
| 570 | 0 | 1 | 15.3 | 214.36 | 99.43 | 266.34 | 31.82 | 2013 |
| 571 | 0 | 2 | 12.6 | 222.81 | 97.04 | 268.97 | 31.08 | 2013 |
| 572 | 0 | 3 | 7.2 | 222.81 | 97.04 | 268.97 | 31.08 | 2013 |
| 573 | 0 | 3 | 8.1 | 220.38 | 96.51 | 266.97 | 30.93 | 2013 |
| 574 | 0 | 1 | 14.4 | 220.38 | 96.51 | 266.97 | 30.93 | 2013 |
| 575 | 0 | 3 | 8.1 | 206.23 | 97.08 | 253.07 | 31.14 | 2013 |
| 576 | 0 | 1 | 7.2 | 193.71 | 96.41 | 240.98 | 30.98 | 2013 |
| 577 | 0 | 3 | 8.1 | 225.22 | 93.33 | 272.44 | 29.97 | 2013 |
| 581 | 0 | 2 | 5.4 | 11.71 | 131.99 | 59.81 | 42.59 | 2013 |
| 582 | 0 | 2 | 9 | 153.89 | 95.73 | 194.28 | 31.09 | 2013 |
| 584 | 0 | 1 | 9.9 | 161.77 | 94.88 | 201.14 | 30.84 | 2013 |
| 586 | 0 | 3 | 4.5 | 142.39 | 96.79 | 182.93 | 31.45 | 2013 |
| 587 | 0 | 2 | 7.2 | 142.39 | 96.79 | 182.93 | 31.45 | 2013 |
| 588 | 0 | 3 | 7.2 | 127.96 | 98.07 | 168.08 | 31.89 | 2013 |
| 589 | 0 | 1 | 3.6 | 127.96 | 98.07 | 168.08 | 31.89 | 2013 |
| 590 | 0 | 1 | 3.6 | 127.96 | 98.07 | 168.08 | 31.89 | 2013 |
| 591 | 0 | 3 | 10.8 | 149.32 | 106.44 | 190.41 | 34.17 | 2013 |
| 593 | 0 | 3 | 5.3 | 149.32 | 106.44 | 190.41 | 34.17 | 2013 |
| 595 | 0 | 3 | 7.2 | 132.40 | 109.33 | 170.38 | 35.13 | 2013 |
| 596 | 0 | 2 | 12.6 | 132.40 | 109.33 | 170.38 | 35.13 | 2013 |
| 597 | 0 | 1 | 9.9 | 132.40 | 109.33 | 170.38 | 35.13 | 2013 |
| 598 | 0 | 3 | 5.4 | 102.48 | 114.76 | 127.15 | 37.05 | 2013 |
| 599 | 0 | 2 | 10.8 | 102.48 | 114.76 | 127.15 | 37.05 | 2013 |
| 600 | 0 | 2 | 10.8 | 102.48 | 114.76 | 127.15 | 37.05 | 2013 |
| 601 | 0 | 2 | 11 | 102.48 | 114.76 | 127.15 | 37.05 | 2013 |
| 602 | 0 | 2 | 12.6 | 5.77 | 134.69 | 53.96 | 43.78 | 2013 |
| 603 | 0 | 2 | 15.3 | 5.77 | 134.69 | 53.96 | 43.78 | 2013 |
| 604 | 0 | 1 | 5.4 | 10.96 | 110.18 | 42.61 | 36.10 | 2013 |
| 604 | 0 | 1 | 7.2 | 10.96 | 110.18 | 42.61 | 36.10 | 2013 |
| 605 | 0 | 1 | 3.6 | 14.37 | 109.97 | 42.37 | 36.05 | 2013 |
| 605 | 0 | 1 | 8.1 | 14.37 | 109.97 | 42.37 | 36.05 | 2013 |
| 607 | 0 | 2 | 6.3 | 20.42 | 111.78 | 20.42 | 36.76 | 2013 |
| 608 | 0 | 1 | 8.4 | 20.42 | 111.78 | 20.42 | 36.76 | 2013 |
| 609 | 0 | 1 | 8.1 | 83.78 | 117.19 | 139.65 | 37.81 | 2013 |
| 610 | 0 | 2 | 7.2 | 83.78 | 117.19 | 139.65 | 37.81 | 2013 |
| 611 | 0 | 2 | 14.4 | 83.78 | 117.19 | 139.65 | 37.81 | 2013 |
| 612 | 0 | 1 | 11.7 | 59.58 | 121.32 | 140.79 | 39.15 | 2013 |
| 613 | 0 | 1 | 9.9 | 55.58 | 122.01 | 134.44 | 39.36 | 2013 |
| 614 | 0 | 3 | 7.2 | 55.58 | 122.01 | 134.44 | 39.36 | 2013 |
| 615 | 0 | 2 | 12.6 | 43.82 | 127.59 | 78.06 | 41.63 | 2013 |
| 616 | 0 | 3 | 9 | 43.82 | 127.59 | 78.06 | 41.63 | 2013 |
| 618 | 0 | 1 | 9.9 | 44.50 | 133.23 | 20.90 | 44.04 | 2013 |
| 619 | 0 | 2 | 10.8 | 2.70 | 128.62 | 92.00 | 41.30 | 2013 |
| 621 | 0 | 2 | 19.8 | 0.90 | 135.94 | 33.44 | 44.39 | 2013 |
| 622 | 0 | 1 | 17.8 | 0.90 | 135.94 | 33.44 | 44.39 | 2013 |
| 628 | 0 | 2 | 10.8 | 1.00 | 134.41 | 58.40 | 43.59 | 2013 |
| 629 | 0 | 1 | 10.3 | 0.50 | 134.41 | 58.40 | 43.59 | 2013 |
| 630 | 0 | 1 | 19.8 | 7.59 | 134.41 | 58.40 | 43.59 | 2013 |
| 631 | 0 | 2 | 22.5 | 7.59 | 134.41 | 58.40 | 43.59 | 2013 |
| 632 | 0 | 1 | 13.5 | 0.90 | 137.24 | 9.47 | 45.45 | 2013 |
| 633 | 0 | 1 | 19.8 | 5.40 | 137.24 | 9.47 | 45.45 | 2013 |
| 637 | 0 | 2 | 23.4 | 6.90 | 137.75 | 26.06 | 48.63 | 2013 |
| 638 | 0 | 2 | 8.1 | 8.10 | 137.75 | 26.06 | 48.63 | 2013 |
| 639 | 0 | 2 | 14.4 | 1.80 | 137.75 | 26.06 | 48.63 | 2013 |
| 640 | 0 | 3 | 7.2 | 1.80 | 134.22 | 60.11 | 43.55 | 2013 |
| 642 | 0 | 1 | 5.4 | 3.60 | 128.50 | 98.60 | 41.26 | 2013 |
| 643 | 0 | 2 | 16 | 2.50 | 137.04 | 14.37 | 45.58 | 2013 |
| 646 | 0 | 1 | 10.8 | 2.70 | 136.47 | 27.84 | 45.52 | 2013 |
| 647 | 0 | 1 | 19.8 | 6.30 | 136.47 | 27.84 | 45.52 | 2013 |
| 652 | 0 | 2 | 15.3 | 4.50 | 137.88 | 17.77 | 48.23 | 2013 |
| 653 | 0 | 2 | 4.5 | 7.20 | 138.08 | 26.77 | 49.02 | 2013 |
| 654 | 0 | 1 | 9 | 2.70 | 138.08 | 26.77 | 49.02 | 2013 |
| 656 | 0 | 2 | 23.4 | 10.80 | 135.71 | 12.58 | 50.02 | 2013 |
| 658 | 0 | 2 | 16.2 | 2.70 | 135.72 | 9.03 | 49.38 | 2013 |
| 659 | 0 | 3 | 19.8 | 6.30 | 135.72 | 9.03 | 49.37 | 2013 |
| 660 | 1 | 2 | 30.6 | 12.70 | 135.72 | 9.03 | 49.36 | 2013 |
| 662 | 0 | 1 | 9 | 7.20 | 134.03 | 14.37 | 50.16 | 2013 |
| 663 | 0 | 3 | 5.4 | 10.80 | 134.03 | 14.37 | 50.16 | 2013 |
| 664 | 0 | 2 | 16.2 | 1.00 | 134.03 | 14.37 | 50.16 | 2013 |
| 665 | 0 | 2 | 10.8 | 1.00 | 130.06 | 78.74 | 41.81 | 2013 |
| 667 | 0 | 2 | 17.1 | 8.10 | 133.77 | 19.95 | 50.83 | 2013 |
| 669 | 0 | 2 | 16.2 | 0.09 | 137.86 | 33.88 | 47.27 | 2013 |
| 671 | 0 | 2 | 8.1 | 0.09 | 137.86 | 33.88 | 47.27 | 2013 |
| 673 | 0 | 1 | 23.4 | 9.00 | 137.54 | 26.52 | 49.52 | 2013 |
| 674 | 0 | 1 | 14 | 0.40 | 137.54 | 26.52 | 49.52 | 2013 |
| 678 | 0 | 3 | 23.4 | 0.90 | 122.97 | 6.96 | 50.76 | 2013 |
| 679 | 0 | 2 | 16.2 | 3.60 | 122.97 | 6.96 | 50.76 | 2013 |
| 680 | 0 | 3 | 16 | 3.80 | 122.97 | 6.96 | 50.76 | 2013 |
| 683 | 0 | 2 | 18 | 3.60 | 121.04 | 13.06 | 50.15 | 2013 |
| 684 | 0 | 2 | 19.8 | 5.40 | 121.04 | 13.06 | 50.15 | 2013 |
| 685 | 0 | 2 | 22.5 | 8.10 | 121.04 | 13.06 | 50.15 | 2013 |
| 688 | 0 | 2 | 21.6 | 0.80 | 129.95 | 42.75 | 48.48 | 2013 |
| 689 | 0 | 3 | 15.3 | 1.80 | 129.95 | 42.75 | 48.48 | 2013 |
| 693 | 0 | 1 | 9.9 | 3.10 | 127.57 | 79.27 | 46.78 | 2013 |
| 694 | 0 | 2 | 13 | 1.00 | 127.57 | 79.27 | 46.78 | 2013 |
| 695 | 0 | 3 | 19.8 | 6.80 | 127.57 | 79.27 | 46.78 | 2013 |
| 696 | 0 | 3 | 13.5 | 0.50 | 127.57 | 79.27 | 46.78 | 2013 |
| 698 | 0 | 2 | 9 | 2.60 | 129.38 | 91.23 | 41.57 | 2013 |
| 699 | 0 | 3 | 16.2 | 8.10 | 129.38 | 91.23 | 41.57 | 2013 |
| 700 | 0 | 2 | 7.2 | 9.00 | 132.07 | 32.17 | 48.31 | 2013 |
| 701 | 0 | 2 | 13.5 | 2.70 | 132.07 | 32.17 | 48.31 | 2013 |
| 702 | 0 | 2 | 18 | 0.09 | 130.62 | 71.64 | 46.67 | 2013 |
| 704 | 0 | 1 | 11.7 | 2.70 | 113.31 | 8.10 | 36.41 | 2013 |
| 705 | 1 | 2 | 19.8 | 1.30 | 113.31 | 134.85 | 36.40 | 2013 |
| 707 | 0 | 1 | 18 | 37.76 | 130.47 | 93.70 | 42.39 | 2013 |
| 708 | 0 | 3 | 10.8 | 0.09 | 120.37 | 41.72 | 49.51 | 2013 |
| 710 | 0 | 3 | 10.8 | 0.13 | 124.24 | 57.16 | 49.07 | 2013 |
| 711 | 0 | 1 | 13.5 | 0.13 | 124.24 | 57.16 | 49.07 | 2013 |
| 713 | 0 | 2 | 9 | 1.80 | 131.90 | 80.78 | 47.17 | 2013 |
| 714 | 0 | 3 | 4.5 | 6.30 | 131.90 | 80.78 | 47.17 | 2013 |
| 715 | 0 | 3 | 10.8 | 2.70 | 129.97 | 61.40 | 47.86 | 2013 |
| 717 | 0 | 2 | 8 | 3.00 | 130.28 | 78.71 | 41.89 | 2013 |
| 721 | 0 | 1 | 16.3 | 1.00 | 134.81 | 13.76 | 49.95 | 2013 |
| 722 | 0 | 3 | 18 | 1.00 | 134.81 | 13.76 | 49.95 | 2013 |
| 723 | 0 | 2 | 25 | 7.00 | 134.81 | 13.76 | 49.95 | 2013 |
| 724 | 0 | 2 | 10.8 | 3.60 | 134.81 | 13.76 | 49.95 | 2013 |
| 726 | 0 | 3 | 9 | 3.60 | 135.38 | 24.80 | 49.27 | 2013 |
| 727 | 0 | 1 | 21.6 | 1.00 | 135.77 | 2.10 | 48.93 | 2013 |
| 728 | 0 | 1 | 22.6 | 1.00 | 135.77 | 3.10 | 48.93 | 2013 |
| 729 | 0 | 3 | 11.7 | 3.60 | 135.77 | 7.80 | 48.94 | 2013 |
| 730 | 0 | 1 | 14.4 | 5.40 | 135.77 | 5.10 | 48.94 | 2013 |
| 731 | 0 | 2 | 14.4 | 5.40 | 135.77 | 5.10 | 48.94 | 2013 |
| 732 | 0 | 1 | 15.4 | 4.40 | 135.77 | 4.10 | 48.93 | 2013 |
| 733 | 1 | 3 | 19.5 | 0.30 | 135.77 | 1.00 | 48.93 | 2013 |
| 737 | 0 | 1 | 13.4 | 0.30 | 137.71 | 18.55 | 50.08 | 2013 |
| 738 | 0 | 2 | 9 | 10.80 | 137.71 | 18.94 | 50.00 | 2013 |
| 739 | 0 | 3 | 9 | 10.80 | 137.71 | 18.94 | 50.00 | 2013 |
| 740 | 0 | 1 | 19.8 | 0.90 | 137.71 | 18.94 | 50.00 | 2013 |
| 743 | 0 | 2 | 13.5 | 2.70 | 112.87 | 25.11 | 37.48 | 2013 |
| 745 | 0 | 1 | 12.5 | 0.10 | 131.06 | 71.30 | 42.19 | 2013 |
| 746 | 0 | 1 | 19.8 | 7.20 | 131.06 | 71.30 | 42.19 | 2013 |
| 747 | 0 | 1 | 18 | 7.20 | 127.16 | 36.97 | 51.27 | 2013 |
| 748 | 0 | 1 | 12.6 | 1.80 | 127.16 | 36.97 | 51.27 | 2013 |
| 750 | 0 | 3 | 12.6 | 2.70 | 130.17 | 16.20 | 49.45 | 2013 |
| 751 | 0 | 3 | 16.2 | 6.30 | 130.17 | 16.20 | 49.45 | 2013 |
| 752 | 0 | 1 | 9 | 0.90 | 130.17 | 16.20 | 49.45 | 2013 |
| 753 | 0 | 3 | 17.1 | 7.20 | 130.17 | 16.20 | 49.45 | 2013 |
| 754 | 0 | 2 | 9 | 3.60 | 118.55 | 14.84 | 47.85 | 2013 |
| 755 | 0 | 3 | 14.4 | 2.94 | 137.94 | 11.71 | 49.90 | 2013 |
| 756 | 0 | 3 | 17.1 | 0.90 | 137.94 | 11.71 | 49.90 | 2013 |
| 757 | 0 | 3 | 16 | 2.00 | 137.94 | 11.71 | 49.90 | 2013 |
| 760 | 0 | 3 | 5.7 | 4.73 | 130.06 | 65.04 | 47.76 | 2013 |
| 761 | 0 | 3 | 13.5 | 2.70 | 130.06 | 65.04 | 47.76 | 2013 |
| 766 | 0 | 2 | 9.9 | 2.70 | 135.17 | 48.27 | 44.00 | 2013 |
| 767 | 0 | 3 | 10.8 | 3.60 | 137.57 | 7.41 | 45.98 | 2013 |
| 769 | 0 | 2 | 18 | 1.00 | 126.13 | 74.11 | 41.66 | 2013 |
| 770 | 0 | 3 | 16 | 0.20 | 134.28 | 6.96 | 50.61 | 2013 |
| 771 | 0 | 1 | 17.5 | 0.40 | 124.51 | 26.51 | 50.86 | 2013 |
| 773 | 0 | 2 | 12.6 | 4.50 | 124.51 | 26.51 | 50.86 | 2013 |
| 774 | 0 | 2 | 12.8 | 4.30 | 124.51 | 26.51 | 50.86 | 2013 |
| 775 | 0 | 2 | 12.6 | 4.50 | 127.18 | 23.96 | 51.23 | 2013 |
| 777 | 0 | 1 | 30.6 | 13.03 | 127.18 | 23.96 | 51.23 | 2013 |
| 778 | 0 | 1 | 12.6 | 1.00 | 136.05 | 36.98 | 44.43 | 2013 |
| 779 | 0 | 2 | 21.6 | 1.80 | 136.05 | 36.98 | 44.43 | 2013 |
| 780 | 0 | 1 | 10 | 7.56 | 125.01 | 70.19 | 48.27 | 2013 |
| 781 | 0 | 2 | 15.8 | 2.20 | 125.01 | 70.19 | 48.27 | 2013 |
| 782 | 0 | 2 | 18 | 7.20 | 122.93 | 43.85 | 50.00 | 2013 |
| 783 | 0 | 2 | 19.8 | 5.40 | 122.93 | 43.85 | 50.00 | 2013 |
| 784 | 0 | 2 | 23.4 | 1.80 | 122.93 | 43.85 | 50.00 | 2013 |
| 785 | 0 | 3 | 30 | 4.80 | 122.93 | 43.85 | 50.00 | 2013 |
| 787 | 0 | 1 | 14.4 | 7.20 | 130.65 | 3.99 | 51.03 | 2013 |
| 788 | 0 | 2 | 18 | 3.60 | 130.65 | 3.99 | 51.03 | 2013 |
| 789 | 0 | 1 | 11.7 | 9.90 | 130.65 | 3.99 | 51.03 | 2013 |
| 793 | 0 | 2 | 21.5 | 10.80 | 130.61 | 26.32 | 50.97 | 2013 |
| 795 | 0 | 2 | 28 | 6.40 | 137.08 | 31.81 | 45.45 | 2013 |
| 796 | 0 | 2 | 29 | 7.40 | 137.08 | 31.81 | 45.45 | 2013 |
| 797 | 0 | 1 | 14.7 | 1.50 | 137.08 | 31.81 | 45.45 | 2013 |
| 798 | 0 | 1 | 27 | 5.40 | 137.08 | 31.81 | 45.45 | 2013 |
| 799 | 0 | 1 | 25.2 | 3.60 | 137.08 | 31.81 | 45.45 | 2013 |
| 800 | 0 | 1 | 27.9 | 6.30 | 137.08 | 31.81 | 45.45 | 2013 |
| 2 | 0 | 1 | 9 | 4.42 | 252.51 | 96.83 | 7.48 | 2017 |
| 4 | 0 | 2 | 9 | 12.58 | 250.86 | 120.57 | 7.57 | 2017 |
| 5 | 0 | 3 | 5.4 | 12.58 | 250.86 | 120.57 | 7.57 | 2017 |
| 6 | 0 | 3 | 9.9 | 17.67 | 255.97 | 84.04 | 7.71 | 2017 |
| 8 | 0 | 3 | 15.3 | 1.00 | 211.03 | 121.55 | 6.39 | 2017 |
| 9 | 0 | 2 | 5.4 | 1.00 | 211.03 | 121.55 | 6.39 | 2017 |
| 11 | 0 | 2 | 12.6 | 9.38 | 213.51 | 112.17 | 6.45 | 2017 |
| 12 | 0 | 2 | 12.6 | 18.72 | 218.18 | 107.50 | 6.57 | 2017 |
| 13 | 0 | 1 | 14.4 | 7.98 | 256.42 | 70.44 | 7.54 | 2017 |
| 14 | 0 | 3 | 4.5 | 7.98 | 256.42 | 70.44 | 7.54 | 2017 |
| 15 | 0 | 2 | 4.5 | 7.98 | 256.42 | 70.44 | 7.54 | 2017 |
| 16 | 0 | 2 | 7.2 | 16.36 | 216.82 | 123.21 | 6.52 | 2017 |
| 18 | 0 | 1 | 7.2 | 41.51 | 221.53 | 71.26 | 6.72 | 2017 |
| 19 | 0 | 2 | 10.8 | 41.51 | 221.53 | 71.26 | 6.72 | 2017 |
| 21 | 0 | 2 | 14.4 | 64.37 | 219.33 | 64.37 | 6.73 | 2017 |
| 22 | 0 | 2 | 5.4 | 37.75 | 224.11 | 37.75 | 6.89 | 2017 |
| 23 | 0 | 2 | 7.2 | 26.61 | 226.34 | 26.61 | 6.96 | 2017 |
| 24 | 0 | 2 | 7.2 | 26.61 | 226.34 | 26.61 | 6.96 | 2017 |
| 26 | 0 | 3 | 19.8 | 43.12 | 228.30 | 29.88 | 6.98 | 2017 |
| 27 | 0 | 2 | 6.3 | 31.15 | 236.46 | 32.40 | 7.24 | 2017 |
| 28 | 0 | 1 | 6.3 | 31.15 | 236.46 | 32.40 | 7.24 | 2017 |
| 29 | 0 | 1 | 9 | 1.00 | 256.16 | 63.05 | 7.56 | 2017 |
| 30 | 0 | 2 | 30.6 | 1.00 | 256.16 | 63.05 | 7.56 | 2017 |
| 31 | 0 | 1 | 5.4 | 1.00 | 257.16 | 63.05 | 7.56 | 2017 |
| 32 | 0 | 1 | 6.5 | 30.98 | 236.77 | 32.45 | 7.25 | 2017 |
| 33 | 0 | 2 | 9 | 32.94 | 241.39 | 41.72 | 7.44 | 2017 |
| 34 | 0 | 1 | 9 | 32.94 | 241.39 | 41.72 | 7.44 | 2017 |
| 36 | 0 | 1 | 4.5 | 1.00 | 235.09 | 3.99 | 7.23 | 2017 |
| 37 | 1 | 2 | 10.8 | 3.99 | 234.38 | 18.88 | 7.21 | 2017 |
| 38 | 0 | 1 | 4.5 | 7.36 | 234.51 | 6.96 | 7.22 | 2017 |
| 39 | 0 | 1 | 4.5 | 16.74 | 237.81 | 20.60 | 7.33 | 2017 |
| 40 | 0 | 2 | 9 | 28.76 | 231.07 | 19.17 | 7.08 | 2017 |
| 41 | 0 | 2 | 15.3 | 28.76 | 231.07 | 19.17 | 7.08 | 2017 |
| 42 | 0 | 1 | 6.3 | 28.76 | 231.07 | 19.17 | 7.08 | 2017 |
| 44 | 0 | 2 | 22.5 | 1.00 | 258.83 | 48.24 | 7.62 | 2017 |
| 48 | 0 | 2 | 5.4 | 79.82 | 228.45 | 79.82 | 7.13 | 2017 |
| 49 | 0 | 1 | 5.4 | 79.82 | 228.45 | 79.82 | 7.13 | 2017 |
| 54 | 0 | 2 | 9 | 37.79 | 241.15 | 110.62 | 7.54 | 2017 |
| 56 | 0 | 3 | 11.7 | 23.34 | 232.33 | 150.30 | 7.30 | 2017 |
| 57 | 0 | 1 | 10.8 | 34.25 | 231.95 | 148.17 | 7.29 | 2017 |
| 60 | 0 | 1 | 6.3 | 44.80 | 234.41 | 132.88 | 7.35 | 2017 |
| 62 | 0 | 1 | 12.6 | 53.53 | 232.77 | 141.42 | 7.30 | 2017 |
| 63 | 0 | 1 | 10.8 | 56.54 | 231.95 | 145.90 | 7.27 | 2017 |
| 64 | 0 | 1 | 16.2 | 55.09 | 231.74 | 147.20 | 7.24 | 2017 |
| 66 | 0 | 1 | 16.8 | 39.04 | 235.22 | 127.14 | 7.27 | 2017 |
| 67 | 0 | 2 | 18.8 | 43.38 | 235.06 | 128.18 | 7.24 | 2017 |
| 68 | 0 | 1 | 10.8 | 48.43 | 235.16 | 128.25 | 7.22 | 2017 |
| 69 | 0 | 1 | 13.5 | 48.43 | 235.16 | 128.25 | 7.22 | 2017 |
| 70 | 0 | 2 | 8.1 | 34.60 | 237.32 | 116.36 | 7.30 | 2017 |
| 71 | 0 | 2 | 11.7 | 34.60 | 237.32 | 116.36 | 7.30 | 2017 |
| 72 | 0 | 3 | 11.7 | 34.60 | 237.32 | 116.36 | 7.30 | 2017 |
| 73 | 0 | 2 | 12.6 | 18.59 | 259.16 | 50.56 | 7.73 | 2017 |
| 74 | 0 | 1 | 7.2 | 18.59 | 259.16 | 50.56 | 7.73 | 2017 |
| 75 | 0 | 1 | 9.9 | 18.59 | 259.16 | 50.56 | 7.73 | 2017 |
| 77 | 0 | 1 | 10.7 | 18.59 | 259.16 | 50.56 | 7.73 | 2017 |
| 78 | 0 | 2 | 16.2 | 18.59 | 259.16 | 50.56 | 7.73 | 2017 |
| 81 | 0 | 2 | 4.5 | 52.08 | 231.88 | 144.85 | 7.21 | 2017 |
| 82 | 0 | 3 | 12.6 | 52.08 | 231.88 | 144.85 | 7.21 | 2017 |
| 83 | 0 | 1 | 17.1 | 52.08 | 231.88 | 144.85 | 7.21 | 2017 |
| 84 | 0 | 2 | 12.6 | 52.08 | 231.88 | 144.85 | 7.21 | 2017 |
| 85 | 0 | 3 | 13.5 | 52.08 | 231.88 | 144.85 | 7.21 | 2017 |
| 86 | 0 | 1 | 9 | 52.78 | 231.84 | 145.73 | 7.23 | 2017 |
| 87 | 0 | 2 | 14.4 | 52.78 | 231.84 | 145.73 | 7.23 | 2017 |
| 88 | 0 | 2 | 14.9 | 52.78 | 231.84 | 145.73 | 7.23 | 2017 |
| 92 | 0 | 2 | 16.2 | 11.84 | 241.78 | 92.37 | 7.58 | 2017 |
| 97 | 0 | 2 | 21.6 | 1.00 | 242.38 | 92.51 | 7.56 | 2017 |
| 98 | 0 | 1 | 11.7 | 16.57 | 243.49 | 99.03 | 7.61 | 2017 |
| 99 | 0 | 2 | 8.1 | 16.57 | 243.49 | 99.03 | 7.61 | 2017 |
| 100 | 0 | 2 | 8.5 | 16.57 | 243.49 | 99.03 | 7.61 | 2017 |
| 101 | 0 | 2 | 20.7 | 1.00 | 248.81 | 67.80 | 7.76 | 2017 |
| 102 | 0 | 2 | 18 | 21.67 | 241.77 | 93.98 | 7.51 | 2017 |
| 103 | 0 | 2 | 13.5 | 12.70 | 249.16 | 50.30 | 7.60 | 2017 |
| 105 | 0 | 2 | 18 | 14.92 | 248.80 | 52.08 | 7.58 | 2017 |
| 107 | 0 | 2 | 14.4 | 40.08 | 244.47 | 79.45 | 7.45 | 2017 |
| 108 | 0 | 3 | 16.2 | 39.36 | 245.50 | 74.07 | 6.48 | 2017 |
| 109 | 0 | 3 | 7.2 | 39.36 | 245.50 | 74.07 | 7.48 | 2017 |
| 110 | 0 | 1 | 12.6 | 4.42 | 259.58 | 4.79 | 7.33 | 2017 |
| 111 | 0 | 1 | 13 | 0.13 | 260.58 | 4.79 | 7.33 | 2017 |
| 112 | 0 | 2 | 16.2 | 0.13 | 260.58 | 4.79 | 7.33 | 2017 |
| 113 | 0 | 3 | 9.9 | 0.13 | 260.58 | 4.79 | 7.33 | 2017 |
| 116 | 1 | 3 | 12.6 | 1.86 | 259.61 | 10.31 | 7.34 | 2017 |
| 118 | 0 | 2 | 18.9 | 1.00 | 260.61 | 1.00 | 7.34 | 2017 |
| 120 | 0 | 3 | 17.1 | 8.24 | 263.08 | 54.70 | 7.51 | 2017 |
| 121 | 0 | 2 | 13.5 | 8.24 | 263.08 | 54.70 | 7.51 | 2017 |
| 124 | 0 | 2 | 27 | 1.00 | 263.39 | 55.03 | 7.55 | 2017 |
| 125 | 0 | 1 | 5.4 | 9.72 | 263.12 | 49.59 | 7.52 | 2017 |
| 127 | 0 | 2 | 9 | 60.38 | 248.55 | 114.07 | 7.25 | 2017 |
| 128 | 0 | 3 | 16.2 | 5.77 | 263.43 | 49.45 | 7.55 | 2017 |
| 132 | 0 | 2 | 6.3 | 1.00 | 263.39 | 24.32 | 7.60 | 2017 |
| 135 | 0 | 2 | 11.7 | 34.42 | 242.18 | 72.19 | 7.16 | 2017 |
| 136 | 0 | 1 | 12.6 | 34.42 | 242.18 | 72.19 | 7.16 | 2017 |
| 138 | 0 | 1 | 16.2 | 34.42 | 242.18 | 72.19 | 7.16 | 2017 |
| 141 | 0 | 2 | 15.3 | 40.77 | 238.08 | 77.59 | 6.87 | 2017 |
| 142 | 0 | 1 | 21.6 | 40.77 | 238.08 | 77.59 | 6.87 | 2017 |
| 143 | 0 | 1 | 9.9 | 40.77 | 238.08 | 77.59 | 6.87 | 2017 |
| 144 | 0 | 2 | 21.6 | 40.77 | 238.08 | 77.59 | 6.87 | 2017 |
| 146 | 0 | 2 | 17.1 | 40.58 | 231.17 | 134.22 | 6.94 | 2017 |
| 147 | 0 | 2 | 12.6 | 40.58 | 231.17 | 134.22 | 6.94 | 2017 |
| 148 | 0 | 3 | 15.3 | 61.16 | 232.50 | 121.56 | 6.85 | 2017 |
| 149 | 0 | 2 | 6.3 | 32.17 | 253.97 | 80.52 | 7.38 | 2017 |
| 150 | 0 | 3 | 15.3 | 32.17 | 253.97 | 80.52 | 7.38 | 2017 |
| 151 | 0 | 3 | 7.2 | 20.90 | 229.30 | 60.75 | 6.48 | 2017 |
| 153 | 0 | 2 | 16.2 | 20.90 | 229.30 | 60.75 | 6.48 | 2017 |
| 154 | 0 | 2 | 14.4 | 20.90 | 229.30 | 60.75 | 6.48 | 2017 |
| 155 | 0 | 3 | 10.8 | 29.03 | 230.33 | 58.18 | 6.52 | 2017 |
| 156 | 0 | 3 | 14.4 | 40.08 | 231.30 | 58.18 | 6.57 | 2017 |
| 157 | 0 | 2 | 6.3 | 25.19 | 233.81 | 37.04 | 6.59 | 2017 |
| 159 | 0 | 2 | 2.7 | 1.00 | 239.65 | 7.59 | 6.73 | 2017 |
| 161 | 0 | 2 | 9.9 | 1.00 | 239.65 | 7.59 | 6.73 | 2017 |
| 162 | 0 | 2 | 14.4 | 1.00 | 239.65 | 7.59 | 6.73 | 2017 |
| 163 | 0 | 1 | 15 | 1.00 | 239.65 | 7.59 | 6.73 | 2017 |
| 166 | 0 | 2 | 13.8 | 1.00 | 240.83 | 1.00 | 6.76 | 2017 |
| 167 | 1 | 2 | 18 | 1.00 | 240.83 | 1.00 | 6.76 | 2017 |
| 169 | 0 | 2 | 29.5 | 1.00 | 240.83 | 1.00 | 6.76 | 2017 |
| 170 | 0 | 3 | 9.9 | 7.10 | 242.10 | 7.10 | 6.80 | 2017 |
| 172 | 0 | 2 | 15 | 6.96 | 244.18 | 25.99 | 6.89 | 2017 |
| 174 | 0 | 2 | 25.2 | 1.00 | 243.95 | 21.35 | 6.83 | 2017 |
| 175 | 0 | 1 | 30.6 | 1.00 | 243.95 | 21.35 | 6.83 | 2017 |
| 176 | 0 | 2 | 4.5 | 1.00 | 243.95 | 21.35 | 6.83 | 2017 |
| 178 | 0 | 2 | 12.6 | 39.81 | 252.16 | 92.96 | 7.37 | 2017 |
| 180 | 0 | 2 | 19.8 | 1.00 | 246.24 | 34.30 | 6.89 | 2017 |
| 182 | 0 | 3 | 22 | 1.00 | 246.24 | 34.30 | 6.89 | 2017 |
| 183 | 0 | 2 | 26.1 | 1.00 | 246.24 | 34.30 | 6.89 | 2017 |
| 184 | 0 | 3 | 6.3 | 1.00 | 246.24 | 34.30 | 6.89 | 2017 |
| 187 | 0 | 2 | 9.9 | 4.79 | 246.90 | 39.08 | 6.90 | 2017 |
| 191 | 0 | 1 | 9 | 34.94 | 234.08 | 119.75 | 7.34 | 2017 |
| 193 | 1 | 3 | 16.3 | 1.00 | 251.59 | 1.00 | 7.15 | 2017 |
| 194 | 0 | 1 | 9.9 | 1.00 | 251.59 | 1.00 | 7.15 | 2017 |
| 195 | 0 | 2 | 22.5 | 1.00 | 251.29 | 15.19 | 7.06 | 2017 |
| 197 | 0 | 2 | 8.1 | 1.00 | 251.93 | 9.23 | 7.08 | 2017 |
| 202 | 0 | 3 | 20.7 | 1.00 | 245.19 | 150.07 | 7.30 | 2017 |
| 204 | 0 | 1 | 17.1 | 13.50 | 260.94 | 23.72 | 7.61 | 2017 |
| 206 | 1 | 2 | 23.4 | 9.23 | 254.00 | 1.00 | 7.11 | 2017 |
| 209 | 0 | 2 | 16.2 | 1.00 | 254.91 | 13.38 | 7.12 | 2017 |
| 214 | 0 | 3 | 9 | 1.00 | 256.41 | 21.96 | 7.17 | 2017 |
| 215 | 0 | 2 | 10.8 | 3.99 | 256.19 | 18.94 | 7.17 | 2017 |
| 218 | 0 | 1 | 13.5 | 4.73 | 255.89 | 26.65 | 7.19 | 2017 |
| 220 | 0 | 2 | 18 | 1.00 | 256.89 | 26.65 | 7.19 | 2017 |
| 221 | 0 | 2 | 13.5 | 1.00 | 256.89 | 26.65 | 7.19 | 2017 |
| 222 | 0 | 2 | 4.5 | 1.00 | 256.89 | 26.65 | 7.19 | 2017 |
| 223 | 0 | 1 | 10.8 | 1.00 | 257.10 | 27.51 | 7.21 | 2017 |
| 225 | 0 | 2 | 11.7 | 1.00 | 257.10 | 27.51 | 7.21 | 2017 |
| 226 | 0 | 1 | 10.8 | 34.08 | 257.63 | 43.35 | 7.32 | 2017 |
| 228 | 0 | 1 | 12.6 | 12.82 | 260.31 | 33.28 | 7.57 | 2017 |
| 230 | 0 | 2 | 23.4 | 1.00 | 248.80 | 35.24 | 6.95 | 2017 |
| 231 | 0 | 2 | 10.8 | 1.00 | 249.80 | 35.24 | 6.95 | 2017 |
| 232 | 0 | 3 | 13.5 | 5.77 | 247.65 | 45.60 | 6.90 | 2017 |
| 233 | 0 | 3 | 9 | 5.77 | 247.65 | 45.60 | 6.90 | 2017 |
| 234 | 0 | 3 | 14.4 | 13.92 | 246.56 | 46.25 | 6.87 | 2017 |
| 235 | 0 | 3 | 9 | 13.92 | 246.56 | 46.25 | 6.87 | 2017 |
| 237 | 0 | 1 | 9 | 0.13 | 243.63 | 32.45 | 6.83 | 2017 |
| 239 | 0 | 1 | 4.5 | 0.13 | 244.63 | 32.45 | 6.83 | 2017 |
| 240 | 0 | 3 | 5.4 | 9.23 | 241.44 | 13.03 | 6.75 | 2017 |
| 241 | 0 | 2 | 23.4 | 9.23 | 241.44 | 13.03 | 6.75 | 2017 |
| 242 | 0 | 3 | 12.6 | 9.23 | 241.44 | 13.03 | 6.75 | 2017 |
| 243 | 0 | 3 | 7.2 | 9.03 | 238.81 | 11.96 | 6.71 | 2017 |
| 245 | 0 | 1 | 25.2 | 1.00 | 239.81 | 11.96 | 6.71 | 2017 |
| 246 | 0 | 2 | 27 | 1.00 | 237.48 | 13.70 | 6.68 | 2017 |
| 252 | 0 | 2 | 12.6 | 1.00 | 249.60 | 75.12 | 6.89 | 2017 |
| 253 | 0 | 1 | 23.4 | 1.00 | 249.60 | 75.12 | 6.89 | 2017 |
| 254 | 0 | 2 | 8.1 | 1.00 | 249.60 | 75.12 | 6.89 | 2017 |
| 256 | 0 | 3 | 3.5 | 3.48 | 248.52 | 85.55 | 6.85 | 2017 |
| 257 | 0 | 2 | 5.5 | 3.48 | 248.52 | 85.55 | 6.85 | 2017 |
| 258 | 0 | 2 | 9 | 9.47 | 248.59 | 75.07 | 6.86 | 2017 |
| 259 | 0 | 2 | 16.2 | 9.47 | 248.59 | 75.07 | 6.86 | 2017 |
| 260 | 0 | 2 | 16.2 | 9.47 | 248.59 | 75.07 | 6.86 | 2017 |
| 261 | 0 | 3 | 3.6 | 9.47 | 248.59 | 75.07 | 6.86 | 2017 |
| 262 | 0 | 1 | 12.6 | 9.47 | 248.59 | 75.07 | 6.86 | 2017 |
| 266 | 0 | 2 | 8.1 | 22.84 | 243.55 | 90.72 | 6.74 | 2017 |
| 267 | 0 | 1 | 16.2 | 43.65 | 231.71 | 119.16 | 7.26 | 2017 |
| 269 | 0 | 2 | 7.2 | 15.47 | 248.76 | 53.64 | 6.89 | 2017 |
| 270 | 0 | 1 | 9 | 19.50 | 243.20 | 89.47 | 6.74 | 2017 |
| 271 | 0 | 2 | 2.7 | 19.50 | 243.20 | 89.47 | 6.74 | 2017 |
| 272 | 0 | 2 | 7 | 14.84 | 242.83 | 89.72 | 5.73 | 2017 |
| 274 | 0 | 2 | 10.8 | 1.00 | 229.28 | 58.69 | 6.45 | 2017 |
| 275 | 0 | 2 | 14.4 | 1.00 | 229.28 | 58.69 | 6.45 | 2017 |
| 277 | 0 | 2 | 9.9 | 46.14 | 219.71 | 46.14 | 6.10 | 2017 |
| 278 | 0 | 3 | 9 | 46.14 | 219.71 | 46.14 | 6.10 | 2017 |
| 279 | 0 | 3 | 12.6 | 48.63 | 219.24 | 48.63 | 6.09 | 2017 |
| 282 | 0 | 3 | 4.5 | 1.00 | 229.37 | 60.35 | 6.32 | 2017 |
| 284 | 0 | 1 | 7.2 | 12.35 | 230.77 | 39.00 | 6.37 | 2017 |
| 286 | 0 | 2 | 16.2 | 12.35 | 230.77 | 39.00 | 6.37 | 2017 |
| 287 | 0 | 3 | 2.7 | 12.35 | 230.77 | 39.00 | 6.37 | 2017 |
| 288 | 0 | 3 | 25.2 | 1.00 | 230.23 | 17.77 | 6.40 | 2017 |
| 291 | 1 | 3 | 16.2 | 1.00 | 228.39 | 1.00 | 6.36 | 2017 |
| 292 | 0 | 2 | 27 | 1.00 | 229.39 | 1.00 | 6.36 | 2017 |
| 294 | 0 | 2 | 16.2 | 18.77 | 225.61 | 18.77 | 6.26 | 2017 |
| 295 | 0 | 1 | 22.5 | 18.77 | 225.61 | 18.77 | 6.26 | 2017 |
| 296 | 0 | 2 | 27 | 18.77 | 225.61 | 18.77 | 6.26 | 2017 |
| 297 | 0 | 1 | 13.5 | 32.97 | 223.69 | 32.97 | 6.23 | 2017 |
| 298 | 0 | 1 | 12.6 | 32.97 | 223.69 | 32.97 | 6.23 | 2017 |
| 299 | 0 | 1 | 12.6 | 36.97 | 223.21 | 36.97 | 6.22 | 2017 |
| 300 | 0 | 3 | 19.8 | 36.97 | 223.21 | 36.97 | 6.22 | 2017 |
| 301 | 0 | 1 | 4 | 26.11 | 234.13 | 39.61 | 6.55 | 2017 |
| 302 | 0 | 2 | 5.4 | 28.50 | 232.71 | 28.50 | 6.48 | 2017 |
| 303 | 0 | 3 | 13.5 | 1.00 | 256.81 | 66.41 | 7.44 | 2017 |
| 304 | 0 | 1 | 4 | 32.35 | 234.12 | 32.35 | 6.52 | 2017 |
| 305 | 0 | 1 | 5.4 | 24.49 | 234.05 | 24.49 | 6.50 | 2017 |
| 306 | 0 | 2 | 7.2 | 24.49 | 234.05 | 24.49 | 6.50 | 2017 |
| 310 | 0 | 1 | 4 | 7.98 | 232.35 | 17.40 | 6.44 | 2017 |
| 311 | 0 | 2 | 5.4 | 7.98 | 232.35 | 17.40 | 6.44 | 2017 |
| 314 | 0 | 3 | 23.4 | 1.00 | 231.11 | 50.21 | 6.40 | 2017 |
| 319 | 0 | 2 | 10.8 | 10.98 | 234.16 | 56.24 | 6.46 | 2017 |
| 320 | 0 | 3 | 6.3 | 10.98 | 234.16 | 56.24 | 6.46 | 2017 |
| 321 | 0 | 2 | 20.7 | 13.86 | 234.33 | 60.64 | 6.46 | 2017 |
| 322 | 0 | 1 | 9.9 | 13.86 | 234.33 | 60.64 | 6.46 | 2017 |
| 323 | 0 | 1 | 11.7 | 13.86 | 234.33 | 60.64 | 6.46 | 2017 |
| 325 | 0 | 1 | 17.1 | 1.00 | 236.04 | 68.81 | 6.54 | 2017 |
| 326 | 0 | 1 | 13.5 | 1.00 | 237.04 | 68.81 | 6.54 | 2017 |
| 327 | 0 | 2 | 12.6 | 13.92 | 255.22 | 78.14 | 7.37 | 2017 |
| 330 | 0 | 1 | 4.5 | 13.92 | 255.22 | 78.14 | 7.37 | 2017 |
| 332 | 0 | 2 | 23.4 | 11.22 | 237.63 | 59.58 | 6.57 | 2017 |
| 334 | 0 | 2 | 18 | 1.00 | 239.58 | 63.64 | 6.63 | 2017 |
| 335 | 0 | 1 | 13.5 | 1.00 | 239.58 | 63.64 | 6.63 | 2017 |
| 336 | 0 | 2 | 15.3 | 1.00 | 239.58 | 63.64 | 6.63 | 2017 |
| 337 | 0 | 2 | 27 | 26.21 | 261.35 | 50.20 | 7.52 | 2017 |
| 338 | 0 | 3 | 6.3 | 26.21 | 261.35 | 50.20 | 7.52 | 2017 |
| 339 | 0 | 1 | 16.2 | 26.98 | 260.91 | 57.39 | 7.53 | 2017 |
| 340 | 0 | 1 | 16.2 | 36.54 | 259.61 | 44.98 | 7.57 | 2017 |
| 350 | 0 | 3 | 10.8 | 1.00 | 259.37 | 45.80 | 7.51 | 2017 |
| 352 | 0 | 2 | 15.3 | 7.36 | 262.58 | 34.75 | 7.60 | 2017 |
| 354 | 0 | 1 | 14.4 | 1.00 | 263.58 | 34.75 | 7.60 | 2017 |
| 355 | 0 | 1 | 11.7 | 13.70 | 263.29 | 26.51 | 7.67 | 2017 |
| 356 | 0 | 2 | 5.4 | 13.70 | 263.29 | 26.51 | 7.67 | 2017 |
| 357 | 0 | 3 | 9 | 20.95 | 262.59 | 30.43 | 7.72 | 2017 |
| 358 | 0 | 3 | 18 | 22.22 | 262.97 | 50.11 | 7.69 | 2017 |
| 359 | 0 | 2 | 10.8 | 22.22 | 262.97 | 50.11 | 7.69 | 2017 |
| 360 | 0 | 3 | 20.7 | 22.22 | 262.97 | 50.11 | 7.69 | 2017 |
| 362 | 0 | 2 | 17.4 | 22.22 | 262.97 | 50.11 | 7.69 | 2017 |
| 364 | 0 | 3 | 6.3 | 22.22 | 262.97 | 50.11 | 7.69 | 2017 |
| 367 | 0 | 2 | 8.1 | 8.10 | 261.88 | 54.62 | 7.67 | 2017 |
| 368 | 0 | 2 | 7.2 | 8.10 | 261.88 | 54.62 | 7.67 | 2017 |
| 371 | 0 | 3 | 7.2 | 26.32 | 255.20 | 26.32 | 7.46 | 2017 |
| 372 | 0 | 2 | 3.6 | 26.32 | 255.20 | 26.32 | 7.46 | 2017 |
| 373 | 0 | 3 | 14.4 | 37.08 | 256.89 | 37.08 | 7.50 | 2017 |
| 376 | 0 | 2 | 9 | 39.03 | 255.36 | 39.03 | 7.51 | 2017 |
| 379 | 0 | 2 | 17.1 | 24.61 | 253.90 | 24.61 | 7.68 | 2017 |
| 380 | 0 | 2 | 9 | 24.14 | 255.15 | 24.14 | 7.77 | 2017 |
| 381 | 0 | 2 | 11.7 | 42.87 | 255.07 | 42.87 | 7.80 | 2017 |
| 382 | 0 | 2 | 11.7 | 19.65 | 257.56 | 19.65 | 7.76 | 2017 |
| 383 | 0 | 1 | 13.5 | 19.65 | 257.56 | 19.65 | 7.76 | 2017 |
| 384 | 0 | 1 | 12.6 | 39.36 | 261.76 | 63.16 | 7.74 | 2017 |
| 385 | 0 | 3 | 8.1 | 39.36 | 261.76 | 63.16 | 7.74 | 2017 |
| 386 | 0 | 2 | 15.3 | 41.57 | 261.18 | 60.64 | 7.76 | 2017 |
| 387 | 0 | 2 | 12.6 | 41.57 | 261.18 | 60.64 | 7.76 | 2017 |
| 388 | 0 | 2 | 9.9 | 48.63 | 261.09 | 64.34 | 7.77 | 2017 |
| 389 | 0 | 2 | 4.5 | 48.63 | 261.09 | 64.34 | 7.77 | 2017 |
| 390 | 0 | 3 | 13.5 | 22.22 | 260.19 | 53.54 | 7.44 | 2017 |
| 394 | 0 | 2 | 9.9 | 60.50 | 259.11 | 71.46 | 7.80 | 2017 |
| 395 | 0 | 2 | 15.3 | 60.50 | 259.11 | 71.46 | 7.80 | 2017 |
| 397 | 0 | 1 | 7.2 | 60.50 | 259.11 | 71.46 | 7.80 | 2017 |
| 398 | 0 | 1 | 8.1 | 60.50 | 259.11 | 71.46 | 7.80 | 2017 |
| 399 | 0 | 2 | 16.2 | 61.81 | 258.88 | 73.55 | 7.80 | 2017 |
| 400 | 0 | 1 | 10.8 | 57.74 | 257.19 | 60.65 | 7.81 | 2017 |
| 401 | 0 | 2 | 12.6 | 57.74 | 257.19 | 60.65 | 7.81 | 2017 |
| 402 | 0 | 1 | 5.4 | 40.79 | 254.61 | 72.51 | 7.80 | 2017 |
| 403 | 0 | 1 | 5.4 | 27.09 | 251.30 | 68.59 | 7.79 | 2017 |
| 404 | 0 | 1 | 8.1 | 27.09 | 251.30 | 68.59 | 7.79 | 2017 |
| 410 | 0 | 2 | 3.6 | 25.43 | 249.80 | 92.74 | 7.65 | 2017 |
| 411 | 0 | 1 | 16.2 | 26.52 | 247.12 | 76.02 | 7.61 | 2017 |
| 412 | 0 | 3 | 18.9 | 23.30 | 245.69 | 66.77 | 7.57 | 2017 |
| 413 | 0 | 1 | 9 | 9.38 | 242.64 | 161.14 | 7.21 | 2017 |
| 415 | 0 | 2 | 16.2 | 1.00 | 253.15 | 93.56 | 7.26 | 2017 |
| 416 | 0 | 2 | 17.1 | 1.00 | 253.15 | 93.56 | 7.26 | 2017 |
| 417 | 0 | 2 | 7.2 | 1.00 | 253.15 | 93.56 | 7.26 | 2017 |
| 418 | 0 | 2 | 9.9 | 14.25 | 255.44 | 81.45 | 7.32 | 2017 |
| 422 | 0 | 1 | 10.8 | 14.25 | 255.44 | 81.45 | 7.32 | 2017 |
| 423 | 0 | 1 | 8.1 | 15.12 | 243.62 | 155.61 | 7.24 | 2017 |
| 425 | 0 | 3 | 7.2 | 12.89 | 261.98 | 17.13 | 7.58 | 2017 |
| 426 | 0 | 2 | 11.7 | 1.00 | 255.01 | 82.28 | 7.30 | 2017 |
| 437 | 0 | 2 | 9.3 | 1.00 | 255.37 | 92.37 | 7.24 | 2017 |
| 438 | 0 | 2 | 10.8 | 1.00 | 255.37 | 92.37 | 7.24 | 2017 |
| 440 | 0 | 1 | 9 | 1.00 | 255.62 | 94.34 | 7.23 | 2017 |
| 442 | 0 | 1 | 5.4 | 1.00 | 255.62 | 94.34 | 7.23 | 2017 |
| 443 | 0 | 2 | 6.3 | 1.00 | 258.63 | 75.30 | 7.34 | 2017 |
| 446 | 0 | 2 | 7.2 | 10.39 | 261.86 | 17.77 | 7.59 | 2017 |
| 447 | 0 | 2 | 13 | 10.39 | 261.86 | 17.77 | 7.59 | 2017 |
| 448 | 0 | 3 | 14.4 | 10.39 | 261.86 | 17.77 | 7.59 | 2017 |
| 455 | 0 | 3 | 12.6 | 1.00 | 262.77 | 54.90 | 7.48 | 2017 |
| 456 | 0 | 1 | 13.5 | 11.22 | 253.39 | 71.87 | 7.01 | 2017 |
| 458 | 0 | 2 | 23.4 | 11.22 | 253.39 | 71.87 | 7.01 | 2017 |
| 463 | 0 | 2 | 18 | 1.00 | 251.54 | 73.23 | 6.95 | 2017 |
| 464 | 0 | 2 | 8.1 | 6.18 | 251.58 | 78.07 | 6.94 | 2017 |
| 469 | 0 | 2 | 11.7 | 31.15 | 261.46 | 47.34 | 7.47 | 2017 |
| 472 | 0 | 2 | 15.3 | 1.00 | 239.96 | 121.01 | 6.60 | 2017 |
| 474 | 0 | 2 | 14 | 1.00 | 238.63 | 116.33 | 6.56 | 2017 |
| 475 | 0 | 3 | 15 | 1.00 | 238.63 | 116.33 | 6.56 | 2017 |
| 480 | 0 | 1 | 10.8 | 1.00 | 231.82 | 85.92 | 6.38 | 2017 |
| 481 | 0 | 3 | 9 | 11.53 | 233.24 | 76.60 | 6.42 | 2017 |
| 482 | 0 | 1 | 3.6 | 11.53 | 233.24 | 76.60 | 6.42 | 2017 |
| 490 | 0 | 3 | 21.6 | 34.33 | 251.80 | 34.33 | 7.59 | 2017 |
| 494 | 1 | 3 | 8.1 | 1.00 | 230.97 | 1.00 | 7.12 | 2017 |
| 495 | 1 | 3 | 12.6 | 1.00 | 230.97 | 1.00 | 7.12 | 2017 |
| 496 | 0 | 2 | 23.4 | 1.00 | 261.54 | 12.58 | 7.38 | 2017 |
| 498 | 0 | 3 | 9.9 | 1.00 | 261.54 | 12.58 | 7.38 | 2017 |
| 499 | 1 | 3 | 16.2 | 16.49 | 231.02 | 16.49 | 7.10 | 2017 |
| 500 | 0 | 3 | 13.5 | 51.17 | 233.86 | 51.17 | 7.27 | 2017 |
| 501 | 0 | 3 | 12.6 | 34.40 | 224.20 | 34.68 | 6.20 | 2017 |
| 502 | 0 | 1 | 5.4 | 62.32 | 234.09 | 66.83 | 7.29 | 2017 |
| 503 | 0 | 2 | 4.5 | 12.11 | 238.31 | 105.62 | 7.20 | 2017 |
| 504 | 0 | 2 | 18 | 12.58 | 261.80 | 31.16 | 7.39 | 2017 |
| 505 | 0 | 2 | 3.6 | 12.58 | 261.80 | 31.16 | 7.39 | 2017 |
| 506 | 0 | 2 | 10.8 | 12.58 | 261.80 | 31.16 | 7.39 | 2017 |
| 507 | 0 | 1 | 14.4 | 20.11 | 246.96 | 31.72 | 7.00 | 2017 |
| 508 | 0 | 1 | 9 | 7.35 | 246.42 | 39.23 | 6.88 | 2017 |
| 509 | 0 | 1 | 5.4 | 12.74 | 248.00 | 42.79 | 6.92 | 2017 |
| 510 | 0 | 3 | 6.3 | 8.46 | 241.62 | 75.57 | 6.69 | 2017 |
| 511 | 0 | 3 | 3.6 | 24.26 | 259.70 | 43.02 | 7.28 | 2017 |
| 512 | 0 | 2 | 2.7 | 24.53 | 258.91 | 57.93 | 7.43 | 2017 |
| 513 | 0 | 3 | 4.5 | 24.53 | 258.91 | 57.93 | 7.43 | 2017 |
| 514 | 0 | 2 | 7.2 | 30.49 | 255.47 | 69.90 | 7.44 | 2017 |
| 515 | 0 | 2 | 4.5 | 51.39 | 229.45 | 216.93 | 6.80 | 2017 |
| 516 | 0 | 2 | 7.2 | 51.39 | 229.45 | 216.93 | 6.80 | 2017 |
| 518 | 0 | 1 | 6.3 | 31.15 | 235.86 | 192.06 | 6.97 | 2017 |
| 519 | 0 | 2 | 9 | 13.68 | 259.56 | 45.65 | 7.75 | 2017 |
| 523 | 0 | 3 | 8.1 | 3.99 | 246.54 | 138.37 | 7.32 | 2017 |
| 524 | 0 | 3 | 17.1 | 3.99 | 246.54 | 138.37 | 7.32 | 2017 |
| 525 | 0 | 2 | 9.9 | 3.99 | 246.54 | 138.37 | 7.32 | 2017 |
| 527 | 0 | 3 | 9 | 60.84 | 230.01 | 219.71 | 6.77 | 2017 |
| 528 | 0 | 3 | 11.7 | 27.50 | 234.15 | 205.15 | 6.94 | 2017 |
| 529 | 0 | 2 | 16.2 | 103.58 | 221.13 | 263.66 | 6.48 | 2017 |
| 530 | 0 | 2 | 9.9 | 67.29 | 211.13 | 291.47 | 6.21 | 2017 |
| 531 | 0 | 2 | 10.8 | 64.16 | 211.76 | 288.12 | 6.23 | 2017 |
| 532 | 0 | 2 | 18 | 62.38 | 212.77 | 285.21 | 6.26 | 2017 |
| 533 | 0 | 3 | 18.9 | 62.38 | 212.77 | 285.21 | 6.26 | 2017 |
| 535 | 0 | 1 | 5.4 | 62.95 | 214.16 | 282.94 | 6.30 | 2017 |
| 536 | 0 | 2 | 9 | 43.93 | 224.19 | 233.62 | 6.63 | 2017 |
| 537 | 0 | 3 | 2.7 | 43.93 | 224.19 | 233.62 | 6.63 | 2017 |
| 540 | 0 | 3 | 19.8 | 9.03 | 245.83 | 136.16 | 6.33 | 2017 |
| 542 | 0 | 2 | 10.8 | 19.45 | 237.01 | 184.69 | 7.05 | 2017 |
| 544 | 0 | 2 | 12.6 | 33.75 | 238.85 | 161.22 | 7.14 | 2017 |
| 545 | 0 | 3 | 18 | 8.10 | 220.02 | 208.74 | 6.53 | 2017 |
| 548 | 0 | 1 | 9.9 | 28.99 | 258.75 | 58.10 | 7.76 | 2017 |
| 549 | 0 | 2 | 8.1 | 11.96 | 248.78 | 124.55 | 7.37 | 2017 |
| 549 | 0 | 3 | 6.3 | 11.96 | 248.78 | 124.55 | 7.37 | 2017 |
| 550 | 0 | 2 | 16.2 | 11.96 | 248.78 | 124.55 | 7.37 | 2017 |
| 551 | 0 | 2 | 14.4 | 27.50 | 258.59 | 58.70 | 7.75 | 2017 |
| 552 | 0 | 1 | 6.3 | 39.30 | 197.13 | 321.68 | 5.79 | 2017 |
| 554 | 0 | 2 | 14.4 | 33.56 | 196.16 | 327.36 | 5.76 | 2017 |
| 555 | 0 | 1 | 7.2 | 21.96 | 194.53 | 339.42 | 5.71 | 2017 |
| 556 | 0 | 1 | 7.2 | 14.99 | 193.27 | 346.03 | 5.67 | 2017 |
| 557 | 0 | 1 | 7.8 | 14.99 | 193.27 | 346.03 | 5.67 | 2017 |
| 559 | 0 | 2 | 2.5 | 1.00 | 190.78 | 360.88 | 5.59 | 2017 |
| 560 | 0 | 3 | 3.5 | 1.00 | 190.78 | 360.88 | 5.59 | 2017 |
| 561 | 0 | 3 | 7.2 | 11.22 | 187.94 | 367.91 | 5.54 | 2017 |
| 562 | 0 | 2 | 7.2 | 1.00 | 188.94 | 367.91 | 5.54 | 2017 |
| 563 | 0 | 2 | 10.8 | 1.00 | 188.94 | 367.91 | 5.54 | 2017 |
| 565 | 0 | 3 | 23.4 | 25.65 | 190.20 | 350.06 | 5.58 | 2017 |
| 566 | 0 | 2 | 7 | 25.65 | 190.20 | 350.06 | 5.58 | 2017 |
| 567 | 0 | 2 | 13.5 | 13.30 | 247.43 | 137.04 | 7.36 | 2017 |
| 568 | 0 | 3 | 4.5 | 21.22 | 186.28 | 374.25 | 5.46 | 2017 |
| 569 | 0 | 2 | 4.5 | 21.22 | 186.28 | 374.25 | 5.46 | 2017 |
| 570 | 0 | 1 | 15.3 | 36.06 | 186.96 | 362.10 | 5.49 | 2017 |
| 574 | 0 | 1 | 14.4 | 81.57 | 181.51 | 375.64 | 5.33 | 2017 |
| 577 | 0 | 3 | 8.1 | 130.26 | 175.61 | 392.59 | 5.16 | 2017 |
| 578 | 0 | 1 | 11.8 | 130.26 | 175.61 | 392.59 | 5.16 | 2017 |
| 579 | 0 | 2 | 12.6 | 98.33 | 181.73 | 350.84 | 5.35 | 2017 |
| 580 | 0 | 3 | 13 | 102.98 | 181.84 | 352.02 | 5.36 | 2017 |
| 583 | 0 | 2 | 5.4 | 10.27 | 180.09 | 328.39 | 5.33 | 2017 |
| 585 | 0 | 3 | 4.5 | 1.00 | 177.57 | 335.60 | 5.28 | 2017 |
| 586 | 0 | 3 | 4.5 | 20.44 | 181.00 | 316.94 | 5.39 | 2017 |
| 587 | 0 | 2 | 7.2 | 1.00 | 182.00 | 316.94 | 5.39 | 2017 |
| 589 | 0 | 1 | 3.6 | 14.92 | 184.23 | 302.21 | 5.46 | 2017 |
| 590 | 0 | 1 | 3.6 | 14.92 | 184.23 | 302.21 | 5.46 | 2017 |
| 591 | 0 | 3 | 10.8 | 89.07 | 199.53 | 281.36 | 5.89 | 2017 |
| 592 | 0 | 1 | 10 | 89.07 | 199.53 | 281.36 | 5.89 | 2017 |
| 593 | 0 | 3 | 5.3 | 89.07 | 199.53 | 281.36 | 5.89 | 2017 |
| 594 | 0 | 1 | 10.8 | 76.93 | 204.72 | 253.96 | 6.05 | 2017 |
| 596 | 0 | 2 | 12.6 | 76.93 | 204.72 | 253.96 | 6.05 | 2017 |
| 597 | 0 | 1 | 9.9 | 76.93 | 204.72 | 253.96 | 6.05 | 2017 |
| 599 | 0 | 2 | 10.8 | 26.61 | 214.34 | 194.42 | 6.37 | 2017 |
| 600 | 0 | 2 | 10.8 | 26.61 | 214.34 | 194.42 | 6.37 | 2017 |
| 601 | 0 | 2 | 11 | 26.61 | 214.34 | 194.42 | 6.37 | 2017 |
| 604 | 0 | 1 | 7.2 | 42.54 | 205.61 | 174.95 | 6.15 | 2017 |
| 605 | 0 | 1 | 3.6 | 40.80 | 205.23 | 175.65 | 6.14 | 2017 |
| 605 | 0 | 1 | 8.1 | 40.80 | 205.23 | 175.65 | 6.14 | 2017 |
| 606 | 0 | 2 | 10.8 | 20.42 | 208.46 | 154.26 | 6.25 | 2017 |
| 608 | 0 | 1 | 8.4 | 20.42 | 208.46 | 154.26 | 6.25 | 2017 |
| 611 | 0 | 2 | 14.4 | 1.00 | 218.79 | 186.65 | 6.51 | 2017 |
| 613 | 0 | 1 | 9.9 | 7.56 | 227.42 | 166.74 | 6.79 | 2017 |
| 614 | 0 | 3 | 7.2 | 7.56 | 227.42 | 166.74 | 6.79 | 2017 |
| 615 | 0 | 2 | 12.6 | 64.99 | 237.40 | 113.46 | 7.16 | 2017 |
| 616 | 0 | 3 | 9 | 64.99 | 237.40 | 113.46 | 7.16 | 2017 |
| 617 | 0 | 1 | 19.8 | 9.58 | 248.38 | 110.12 | 6.56 | 2017 |
| 619 | 0 | 2 | 10.8 | 1.00 | 238.56 | 179.37 | 7.12 | 2017 |
| 620 | 0 | 1 | 4.5 | 1.00 | 239.56 | 179.37 | 7.12 | 2017 |
| 622 | 0 | 1 | 17.8 | 1.00 | 253.63 | 102.63 | 7.56 | 2017 |
| 623 | 0 | 2 | 10.8 | 1.00 | 261.92 | 14.60 | 7.64 | 2017 |
| 624 | 0 | 1 | 10.8 | 8.10 | 242.62 | 162.21 | 7.21 | 2017 |
| 625 | 0 | 2 | 6.4 | 8.10 | 242.62 | 162.21 | 7.21 | 2017 |
| 626 | 0 | 2 | 5.4 | 1.00 | 250.42 | 114.72 | 7.47 | 2017 |
| 627 | 0 | 2 | 10.8 | 7.59 | 250.98 | 116.12 | 7.45 | 2017 |
| 629 | 0 | 1 | 10.3 | 7.59 | 250.98 | 116.12 | 7.45 | 2017 |
| 631 | 0 | 2 | 22.5 | 7.59 | 250.98 | 116.12 | 7.45 | 2017 |
| 633 | 0 | 1 | 19.8 | 1.00 | 256.69 | 65.19 | 7.62 | 2017 |
| 634 | 0 | 2 | 7.2 | 1.00 | 256.81 | 70.17 | 7.57 | 2017 |
| 635 | 0 | 2 | 4.5 | 1.00 | 263.01 | 4.79 | 7.63 | 2017 |
| 636 | 0 | 1 | 13.4 | 1.00 | 262.06 | 16.20 | 7.63 | 2017 |
| 638 | 0 | 2 | 8.1 | 1.00 | 263.17 | 14.25 | 7.61 | 2017 |
| 639 | 0 | 2 | 14.4 | 1.00 | 263.17 | 14.25 | 7.61 | 2017 |
| 641 | 0 | 1 | 9 | 8.10 | 238.54 | 177.66 | 7.11 | 2017 |
| 642 | 0 | 1 | 5.4 | 1.00 | 239.54 | 177.66 | 7.11 | 2017 |
| 643 | 0 | 2 | 16 | 1.00 | 258.00 | 57.70 | 7.60 | 2017 |
| 644 | 0 | 2 | 4.5 | 1.00 | 258.00 | 57.70 | 7.60 | 2017 |
| 645 | 0 | 1 | 15.3 | 1.00 | 257.52 | 58.05 | 7.56 | 2017 |
| 646 | 0 | 1 | 10.8 | 1.00 | 257.52 | 58.05 | 7.56 | 2017 |
| 648 | 0 | 2 | 11.7 | 1.00 | 258.52 | 48.96 | 7.59 | 2017 |
| 649 | 0 | 3 | 18 | 1.00 | 258.52 | 48.96 | 7.59 | 2017 |
| 650 | 0 | 3 | 4.5 | 1.00 | 258.52 | 48.96 | 7.59 | 2017 |
| 651 | 0 | 2 | 21.6 | 1.00 | 262.82 | 1.00 | 7.63 | 2017 |
| 652 | 1 | 2 | 15.3 | 1.00 | 262.82 | 1.00 | 7.63 | 2017 |
| 653 | 0 | 2 | 4.5 | 1.00 | 263.51 | 25.62 | 7.63 | 2017 |
| 655 | 0 | 2 | 3.6 | 1.00 | 263.51 | 25.62 | 7.63 | 2017 |
| 657 | 0 | 3 | 11.7 | 1.00 | 262.51 | 36.12 | 7.44 | 2017 |
| 658 | 0 | 3 | 16.2 | 1.00 | 261.52 | 29.98 | 7.45 | 2017 |
| 659 | 0 | 3 | 19.8 | 1.00 | 262.52 | 29.98 | 7.45 | 2017 |
| 660 | 0 | 2 | 30.6 | 1.00 | 262.52 | 29.98 | 7.45 | 2017 |
| 661 | 0 | 3 | 9.9 | 1.00 | 261.66 | 37.33 | 7.49 | 2017 |
| 662 | 0 | 1 | 9 | 1.00 | 261.00 | 19.94 | 7.34 | 2017 |
| 664 | 0 | 2 | 16.2 | 1.00 | 261.00 | 19.94 | 7.34 | 2017 |
| 665 | 0 | 2 | 10.8 | 1.00 | 242.12 | 166.61 | 7.21 | 2017 |
| 666 | 0 | 2 | 5.4 | 1.00 | 242.12 | 166.61 | 7.21 | 2017 |
| 668 | 1 | 3 | 9.9 | 1.00 | 260.79 | 1.00 | 7.34 | 2017 |
| 670 | 0 | 1 | 14.4 | 1.00 | 261.47 | 9.48 | 7.66 | 2017 |
| 672 | 0 | 2 | 14.4 | 9.72 | 263.53 | 43.54 | 7.58 | 2017 |
| 673 | 0 | 1 | 23.4 | 9.72 | 263.53 | 43.54 | 7.58 | 2017 |
| 674 | 0 | 1 | 14 | 9.72 | 263.53 | 43.54 | 7.58 | 2017 |
| 675 | 0 | 2 | 6.3 | 9.72 | 263.53 | 43.54 | 7.58 | 2017 |
| 676 | 0 | 2 | 9.9 | 9.72 | 263.53 | 43.54 | 7.58 | 2017 |
| 677 | 0 | 3 | 12.6 | 13.03 | 263.20 | 51.89 | 7.54 | 2017 |
| 679 | 0 | 2 | 16.2 | 1.00 | 243.82 | 29.98 | 6.90 | 2017 |
| 681 | 0 | 2 | 12.6 | 1.00 | 238.84 | 36.92 | 6.81 | 2017 |
| 682 | 0 | 3 | 10.8 | 1.00 | 239.84 | 36.92 | 6.81 | 2017 |
| 684 | 0 | 2 | 19.8 | 1.00 | 239.84 | 36.92 | 6.81 | 2017 |
| 685 | 0 | 2 | 22.5 | 1.00 | 239.84 | 36.92 | 6.81 | 2017 |
| 686 | 0 | 1 | 8.1 | 1.00 | 239.84 | 36.92 | 6.81 | 2017 |
| 687 | 0 | 3 | 13.5 | 1.00 | 246.30 | 62.34 | 7.58 | 2017 |
| 689 | 0 | 3 | 15.3 | 1.00 | 247.30 | 62.34 | 7.58 | 2017 |
| 690 | 0 | 1 | 12.6 | 1.00 | 241.80 | 93.01 | 7.47 | 2017 |
| 691 | 0 | 2 | 6.3 | 1.00 | 241.80 | 93.01 | 7.47 | 2017 |
| 692 | 0 | 3 | 12.6 | 1.00 | 241.80 | 93.01 | 7.47 | 2017 |
| 693 | 0 | 1 | 9.9 | 1.00 | 240.86 | 94.15 | 7.54 | 2017 |
| 697 | 0 | 2 | 5.4 | 8.10 | 240.10 | 169.92 | 7.16 | 2017 |
| 699 | 0 | 3 | 16.2 | 1.00 | 241.10 | 169.92 | 7.16 | 2017 |
| 700 | 0 | 2 | 7.2 | 1.00 | 250.25 | 43.87 | 7.69 | 2017 |
| 701 | 0 | 2 | 13.5 | 1.00 | 250.25 | 43.87 | 7.69 | 2017 |
| 702 | 0 | 2 | 18 | 1.00 | 246.25 | 82.77 | 7.69 | 2017 |
| 703 | 0 | 1 | 25.2 | 1.00 | 246.25 | 82.77 | 7.69 | 2017 |
| 704 | 0 | 1 | 11.7 | 1.00 | 211.24 | 134.85 | 6.35 | 2017 |
| 705 | 0 | 2 | 19.8 | 1.00 | 211.24 | 134.85 | 6.35 | 2017 |
| 706 | 0 | 1 | 19.8 | 1.00 | 208.13 | 146.71 | 6.26 | 2017 |
| 709 | 0 | 3 | 16.2 | 0.13 | 247.96 | 87.69 | 6.83 | 2017 |
| 711 | 0 | 1 | 13.5 | 0.13 | 247.96 | 87.69 | 6.83 | 2017 |
| 712 | 0 | 2 | 14.4 | 1.00 | 255.10 | 92.77 | 7.24 | 2017 |
| 716 | 0 | 2 | 11 | 3.99 | 242.57 | 163.86 | 7.22 | 2017 |
| 717 | 0 | 2 | 8 | 3.99 | 242.57 | 163.86 | 7.22 | 2017 |
| 718 | 0 | 2 | 15.3 | 3.99 | 242.57 | 163.86 | 7.22 | 2017 |
| 719 | 0 | 1 | 18.9 | 3.99 | 242.57 | 163.86 | 7.22 | 2017 |
| 720 | 0 | 2 | 14.4 | 17.38 | 242.16 | 68.44 | 7.39 | 2017 |
| 721 | 0 | 1 | 16.3 | 1.00 | 255.68 | 1.00 | 7.66 | 2017 |
| 722 | 1 | 3 | 18 | 1.00 | 255.68 | 1.00 | 7.66 | 2017 |
| 725 | 1 | 2 | 18 | 1.00 | 255.56 | 28.76 | 7.73 | 2017 |
| 728 | 0 | 1 | 22.6 | 1.00 | 258.20 | 13.76 | 7.68 | 2017 |
| 730 | 0 | 1 | 14.4 | 1.00 | 257.20 | 13.76 | 7.68 | 2017 |
| 732 | 0 | 1 | 15.4 | 1.00 | 257.20 | 13.76 | 7.68 | 2017 |
| 734 | 0 | 2 | 20.7 | 1.00 | 260.22 | 45.69 | 7.68 | 2017 |
| 735 | 0 | 3 | 16.2 | 1.00 | 261.22 | 45.69 | 7.68 | 2017 |
| 736 | 0 | 2 | 9.9 | 1.00 | 262.04 | 68.59 | 7.62 | 2017 |
| 737 | 0 | 1 | 13.4 | 1.00 | 263.04 | 68.59 | 7.62 | 2017 |
| 738 | 0 | 2 | 9 | 1.00 | 262.36 | 61.09 | 7.66 | 2017 |
| 739 | 0 | 3 | 9 | 1.00 | 262.36 | 61.09 | 7.66 | 2017 |
| 740 | 0 | 1 | 19.8 | 1.00 | 262.36 | 61.09 | 7.66 | 2017 |
| 741 | 0 | 1 | 20.7 | 1.00 | 262.36 | 61.09 | 7.66 | 2017 |
| 742 | 0 | 1 | 19.5 | 1.00 | 262.36 | 61.09 | 7.66 | 2017 |
| 744 | 0 | 2 | 9 | 1.00 | 227.33 | 70.44 | 6.89 | 2017 |
| 745 | 0 | 1 | 12.5 | 1.00 | 244.04 | 156.35 | 7.27 | 2017 |
| 746 | 0 | 1 | 19.8 | 1.00 | 244.04 | 156.35 | 7.27 | 2017 |
| 749 | 0 | 3 | 3.6 | 19.65 | 224.81 | 66.28 | 6.82 | 2017 |
| 750 | 0 | 3 | 12.6 | 0.13 | 256.21 | 55.64 | 7.11 | 2017 |
| 752 | 0 | 1 | 9 | 0.13 | 256.21 | 55.64 | 7.11 | 2017 |
| 753 | 0 | 3 | 17.1 | 0.13 | 256.21 | 55.64 | 7.11 | 2017 |
| 757 | 0 | 3 | 16 | 1.00 | 263.13 | 62.97 | 7.64 | 2017 |
| 758 | 0 | 3 | 19.8 | 1.00 | 263.13 | 62.97 | 7.64 | 2017 |
| 759 | 0 | 2 | 9.9 | 1.00 | 263.13 | 62.97 | 7.64 | 2017 |
| 762 | 0 | 1 | 8.1 | 1.00 | 262.55 | 68.44 | 7.60 | 2017 |
| 763 | 0 | 1 | 15.3 | 1.00 | 246.10 | 115.30 | 6.80 | 2017 |
| 764 | 0 | 2 | 16.2 | 1.00 | 247.10 | 115.30 | 6.80 | 2017 |
| 765 | 0 | 1 | 3.6 | 1.00 | 247.73 | 132.83 | 7.36 | 2017 |
| 768 | 0 | 3 | 4.5 | 1.00 | 258.96 | 49.26 | 7.65 | 2017 |
| 771 | 0 | 1 | 17.5 | 1.00 | 247.49 | 40.66 | 6.92 | 2017 |
| 772 | 0 | 2 | 24.3 | 1.00 | 248.49 | 40.66 | 6.92 | 2017 |
| 775 | 0 | 2 | 12.6 | 1.00 | 252.05 | 17.82 | 7.06 | 2017 |
| 776 | 0 | 3 | 18.9 | 1.00 | 252.05 | 17.82 | 7.06 | 2017 |
| 777 | 0 | 1 | 30.6 | 1.00 | 252.05 | 17.82 | 7.06 | 2017 |
| 784 | 0 | 2 | 23.4 | 1.00 | 246.42 | 65.85 | 6.83 | 2017 |
| 786 | 0 | 2 | 18 | 1.00 | 246.42 | 65.85 | 6.83 | 2017 |
| 790 | 0 | 3 | 21.6 | 1.00 | 257.23 | 31.16 | 7.20 | 2017 |
| 791 | 0 | 1 | 8.1 | 1.00 | 253.63 | 1.00 | 7.35 | 2017 |
| 792 | 1 | 1 | 13.5 | 1.00 | 253.63 | 1.00 | 7.35 | 2017 |
| 793 | 1 | 2 | 21.5 | 1.00 | 252.63 | 1.00 | 7.35 | 2017 |
| 794 | 0 | 1 | 7.2 | 1.00 | 253.63 | 1.00 | 7.35 | 2017 |
| 795 | 0 | 2 | 28 | 1.00 | 256.33 | 81.63 | 7.67 | 2017 |
| 796 | 0 | 2 | 29 | 1.00 | 256.33 | 81.63 | 7.67 | 2017 |
| 797 | 0 | 1 | 14.7 | 1.00 | 256.33 | 81.63 | 7.67 | 2017 |
| 798 | 0 | 1 | 27 | 1.00 | 256.33 | 81.63 | 7.67 | 2017 |
| 800 | 0 | 1 | 27.9 | 1.00 | 256.33 | 81.63 | 7.67 | 2017 |
| 801 | 1 | 2 | 9.9 | 1.00 | 261.91 | 1.00 | 7.68 | 2017 |
